# Flavonoids and Acid-Hydrolysis derivatives of *Neo*-Clerodane diterpenes from *Teucrium flavum* subsp. *glaucum* as inhibitors of the HIV-1 reverse transcriptase–associated RNase H function

**DOI:** 10.1080/14756366.2021.1887170

**Published:** 2021-03-10

**Authors:** Benedetta Fois, Angela Corona, Enzo Tramontano, Simona Distinto, Elias Maccioni, Rita Meleddu, Pierluigi Caboni, Costantino Floris, Filippo Cottiglia

**Affiliations:** a Department of Life and Environmental Sciences, University of Cagliari, Cittadella Universitaria di Monserrato, Monserrato, Italy; b Istituto di Ricerca Genetica e Biomedica, Consiglio Nazionale delle Ricerche, Monserrato, Italy; c Department of Chemical and Geological Sciences, University of Cagliari, Monserrato, Italy

**Keywords:** HIV, RNase H, reverse transcriptase, *Teucrium flavum*, *neo*-clerodane diterpenes, flavonoids

## Abstract

Bioassay-guided fractionation of the ethyl acetate extract from *Teucrium flavum* subsp. *glaucum*, endowed with inhibitory activity towards the HIV-1 reverse transcriptase–associated RNase H function, led to the isolation of salvigenin (**1**), cirsimaritin (**2**) and cirsiliol (**3**) along with the *neo*-clerodanes teuflavin (**4**) and teuflavoside (**5**). Acid hydrolysis of the inactive teuflavoside provided three undescribed *neo*-clerodanes, flavuglaucins A-C (**7-9**) and one known *neo*-clerodane (**10**). Among all *neo*-clerodanes, flavuglaucin B showed the highest inhibitory activity towards RNase H function with a IC_50_ value of 9.1 μM. Molecular modelling and site-directed mutagenesis analysis suggested that flavuglaucin B binds into an allosteric pocket close to RNase H catalytic site. This is the first report of clerodane diterpenoids endowed with anti-reverse transcriptase activity. *Neo*-clerodanes represent a valid scaffold for the development of a new class of HIV-1 RNase H inhibitors.

## Graphical Abstract

**Figure:**
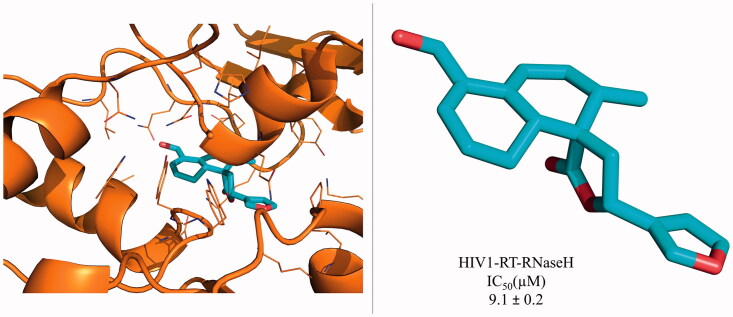

## 1. Introduction

Human Immunodeficiency Virus-1 (HIV-1) is the causative agent of the Acquired Immune Deficiency Syndrome (AIDS). Despite many countries are making progress in lowering AIDS deaths and preventing new infections, in 2019 38 million people were living with HIV^1^. In fact, albeit the Antiretroviral Therapy (ART) has achieved great success in HIV treatment, there is a sub-optimal treatment coverage of infected people (only the 64% according to UNAIDS 2019). The recent fail of pioneering projects of eradication of the infection^2^ or immunisation,^3^ together with raises the number of treatment failures^4^ due to selection and transmission of drug-resistant variants^5–7^, enlight the constant need of finding new drugs with innovative mechanisms of action.

Among the different steps of the viral life cycle identified as drug target, one of the most attracting and explored is represented by the HIV-1 reverse transcriptase (RT). The HIV RT is the enzyme responsible for the reverse transcription of the single-stranded RNA genome into a double-stranded DNA that can eventually integrate in the genome of the infected cell^8^. The RT is a multifunctional enzyme with DNA polymerase (DP) and ribonuclease H (RNase H) activities. All of the currently approved anti-HIV drugs targeting RT inhibit the DP activity. This class can be divided into Nucleoside/Nucleotide RT Inhibitors (NRTIs/NtRTIs) and Non-Nucleoside RT Inhibitors (NNRTIs)^9^. Although also RNase H function is essential for the reverse transcription process^10^, inhibitors that target this enzymatic activity have yet to enter clinical development at any stage. HIV RNase H inhibitors (RNHIs) can be divided in two groups: metal-chelating active site and allosteric inhibitors^10^. The majority of RNHIs are represented by compounds possessing metal coordinating functions such as diketoacids^11^^,^^12^, N-hydroxypyrimidinediones^13^, 2-hydroxyisoquinoline-1,3-diones^14^ and 3,4,5-trihydroxybenzoylhydrazones^15^. Unfortunately, these metal coordinating agents bind many host enzymes catalytic sites leading to toxicity^10^. By contrast, allosteric RNHIs, binding outside the active site, could be more advantageous to avoid the unspecific off-target enzymes inhibition. Among them, both synthetic^16–23^ and natural^24^^,^^25^ molecules have been found to inhibit the RNase H function by selectively targeting allosteric sites.

During the continuous search of secondary plant metabolites with antiviral activity^24–26^, we found that the ethyl acetate (EtOAc) extract from the leaves of *Teucrium flavum* subsp. *glaucum* showed a significant inhibitory activity towards this enzyme function, with an IC_50_ of 28.6 μg/mL (Table 1). In order to identify the molecules responsible of this activity, we decided to carry out a bioguided fractionation of the extract. *T. flavum* subsp. *glaucum* is an evergreen perennial shrub that grows in the calcareous mountains of Sardinia and Corsica islands from sea level up to 1000 m. Previous phytochemical studies of the aerial parts of *T. flavum* subsp. *glaucum* by Savona et al. revealed the presence of *neo*-clerodane diterpenes and flavonoids^27^. However, no biological study on the non-volatile extracts of this plant is reported.

**Table 1.** Results of RT RNase H function inhibition by *T. flavum* subsp. *glaucum* fractions.

Fractions	RNase H
	IC_50_ (µg/mL)^a^
**EtOAc extract**	28.6 ± 3.0
**F1**	25.6 ± 7.4
**F2**	20.3 ± 4.0
**F3**	9.9 ± 1.5
**F4**	>100 (100%)^b^
**F5**	>100 (100%)^b^
**F6**	>100 (100%)^b^

^a^ Concentration capable of inhibiting 50% of enzyme activity.

^b^ Percentage of residual enzyme activity in the presence of 100 µg/mL extract.

## 2. Materials and methods

### 2.1. General experimental procedures

Optical rotations were measured in CHCl_3_ or MeOH at 25 °C using a Perkin-Elmer 241 polarimeter. UV spectra were recorded on a GBC Cintra 5 spectrophotometer. NMR spectra of all isolated compounds were recorded at 25 °C on Unity Inova 500NB high-resolution spectrometer (Agilent Technologies, CA, USA) operating at 500 MHz for ^1^H-NMR and 100 MHz for ^13 ^C-NMR, respectively. Spectra were measured in CDCl_3_ and CD_3_OD and referenced against residual non-deuterated solvents. HRESIMS were measured on an Agilent 6520 Time of Flight (TOF) MS instrument. Column chromatography was carried out under TLC monitoring using silica gel (40–63 µm, Merck), and Sephadex LH-20 (25–100 µm, Pharmacia). For vacuum-liquid chromatography (VLC), silica gel (40– 63 µm) (Merck) was used. TLC was performed on silica gel 60 F_254_ or RP-18 F_254_ (Merck). LiChrolut RP-18 (40–63 μm) 500 mg, 3 mL (Merck) solid phase extraction (SPE) cartridges were also used. Semi-preparative HPLC was conducted by means of a Varian 920 LH instrument fitted with an autosampler module with a 1000 µL loop. The peak purities were monitored using a dual-wavelength UV detector settled at 254 and 360 nm. The columns were a 250 × 10 mm Spherisorb silica, particle size 5 µm (Waters) and a 300 × 7.5 mm Polymeric Reversed Phase (PLRP-S 100 Å), particle size 8 µm (Varian).

### 2.2. Plant material

The leaves of *Teucrium flavum* subsp. *glaucum* were collected in July 2003, at Orgosolo mountains (Sardinia). The plant was identified by Professor Bruno De Martis of the Department of Botanical Sciences of the University of Cagliari. A voucher specimen (No. 0309) was deposited in the Herbarium of the Department of Life and Environmental Science, Drug Sciences Section, University of Cagliari.

### 2.3. Extraction and isolation

Air-dried and powdered leaves of *T. flavum* subsp. *glaucum* (500 g) were ground and extracted with *n*-hexane (3 L) by percolation at room temperature to give 22 g dried extract. The remaining plant material was then extracted with EtOAc (2.5 L), giving 117 g dried extract. The extracts were subsequently stored at −20 °C. A sample of the EtOAc extract was tested in the RT RNase H inhibition assay in March 2019 and then phytochemically investigated.

An aliquot (17 g) of the EtOAc extract was subjected to Vacuum Liquid Chromatography (VLC) (silica gel, 90 g, 40–63 µm) using a step gradient of *n*-hexane/ethyl acetate (7.5: 2.5–0: 10, 500 mL each) to yield 45 fractions. Based on the TLC similarities, identical fractions were combined to give a total of six fractions (F1–F6). Fraction F1 (320 mg) was chromatographed by column chromatography (CC) over Sephadex LH-20 (MeOH) to give compound **1** (10.5 mg). Fraction F2 (900 mg) was subjected to CC over silica gel using DCM/MeOH (9.75: 0.25) as eluent, to furnish three subfractions (F2.1–F2.3). F2.1 was further purified over Sephadex LH-20 (MeOH) to give compound **1** (2.4 mg). F2.2 (55 mg) was chromatographed by Sephadex LH-20 (MeOH) to give a mixture of two compounds (12 mg) that was purified by PLRP HPLC using ACN/H_2_O as eluents (7: 3, flow 2.5 mL/min) to furnish compound **1** (1.5 mg, *t_R_* 12.1 min) and compound **2** (1.9 mg, *t_R_* 8.9 min) . F2.3 (120 mg) was purified using Sephadex LH-20 (MeOH) to give compound **2** (4.4 mg) and a yellow solid (86.8 mg). The obtained solid was purified further by CC over silica gel using DCM/MeOH (9.75: 0.25) as eluent, to give compound **4** (11.3 mg). An aliquot of fraction F3 (200 mg) was chromatographed by CC over Sephadex LH-20 using MeOH as eluent, to furnish an impure compound (16 mg) that was further purified by RP-18 SPE using ACN/H_2_O (5: 5) as eluent, to give compound **3** (3.7 mg). F4 (6.5 g) was subjected to VLC (silica gel, 90 g, 40–63 µm) using a step gradient of DCM/MeOH (9.5: 0.5–8: 2, 500 mL each) to yield 4 subfractions (F4.1–F4.4). F4.3 (4.4 g) was subjected to CC over Sephadex LH-20 using MeOH as eluent giving compound **5** (2.3 g).

### 2.4. Hydrolysis of teuflavoside (5)

Hydrolysis was performed by reacting 800 mg of teuflavoside (**5**) with 5.6 mL of 2 N H_2_SO_4_ in 95 mL of water for 20 min at 95 °C. After cooling, the solution was diluted in water and then extracted with ethyl acetate in a separatory funnel. The organic layer was evaporated by vacuum and the residue (550 mg) was chromatographed over silica gel, using DCM/EtOAc (9.75: 0.25) as eluent giving compound **6** (28.2 mg), **7** (25.1 mg) and two subfractions (F_A_ and F_B_). F_A_ fraction (28.9 mg) was purified by RP-HPLC, using ACN/H_2_O (6: 4, flow 2.5 mL/min), to give compound **9** (1.4 mg, *t_R_* = 10 min), and compound **6** (2.1 *t_R_* = 13 min). F_B_ (10.5 mg) was chromatographed by RP-HPLC, using ACN/H_2_O (6: 4, flow 2.5 mL/min) giving compound **8** (1.5 mg, *t_R_* = 9.5 min), and compound **9** (0.6 mg, *t_R_* = 10 min).

*Flavuglaucin A* (**6**): amorphous solid; [α]^20^_D_ + 48.6 (*c* 0.09, CH_2_Cl_2_); ^1^H (CDCl_3_, 500 MHz) and ^13 ^C (CDCl_3_, 100 MHz) NMR, see Table 2; HRTOFESIMS *m/z* 379.1519 [M + Na]^+^ (calcd for C_21_H_24_O_5_, 379.1515).

**Table 2.** ^1^H NMR and ^13 ^C NMR Spectroscopic Data for Compounds **6**–**9** (CDCl_3_, *δ* in ppm)

Compound **6**			Compound **7**		Compound **8**	
position	δ_C_, type	δ_H_ (*J* in Hz)	δ_C_, type	δ_H_ (*J* in Hz)	δ_C_, type	δ_H_ (*J* in Hz)
1a 1b	24.4, CH_2_	1.53, dddd (13.0, 13.0, 6.0, 5.5) 1.99, m	24.6, CH_2_	1.52, dddd (13.0, 13.0, 6.0, 5.5) 2.0, m	23.3, CH_2_	1.89, m^a^ 2.31, m^a^
2a	25.9, CH_2_	2.29, m^a^	25.8, CH_2_	2.30, m^a^	35.5, CH_2_	2.29, m^a^
2b 3	128.2, CH	5.86, brd	125.8, CH	5.86, brd	197.5, C	2.54, m^a^
4	131.6, C		135.9, C		131.7, C	
5	130.6, C		130.7, C		155.0, C	
6a 6b	121.6, CH	5.77, brd	121.3, CH	5.91, brd	30.5, CH_2_	2.05, dd (13.5, 3.5) 2.99, m^a^
7a 7b	31.7, CH_2_	2.23, m^a^ 2.50, m^a^	31.6, CH_2_	2.26, m^a^ 2.52, m^a^	29.1, CH_2_	1.72, dd (14, 3.5) 2.13, m
8	34.6, CH	1.89, m	34.6, CH	1.89, m	38.9, CH	1.80, m
9	50.4, C		50.6, C		54.5, C	
10	45.2, CH	2.35, m	45.3, CH	2.34, m	46.2, CH	2.49, brd
11	41.3, CH_2_	2.51, m^a^	41.2, CH_2_	2.51, m^a^	40.7, CH_2_	2.51, m^a^
12	71.8, CH	5.46, *t* (8.5)	71.9, CH	5.46, *t* (8.5)	71.8, CH	5.41, *t* (8.5)
13	125.8, C		125.8, C		125.9, C	
14	108.1, CH	6.40, brd	108.1, CH	6.40, brd	107.8, CH	6.40, brd
15	144.0, CH	7.43, *t* (1.5)	144.0, CH	7.43, *t* (1.5)	144.0, CH	7.45, *t* (1.5)
16	139.3, CH	7.45, brd	139.4, CH	7.45, brd	139.3, CH	7.47, brd
17	17.5, CH_3_	1.0, d (6.5)	17.6, CH_3_	1.0, d (6.5)	17.0, CH_3_	1.03, d (6.5)
18a 18b	65.3, CH_2_	4.61, d (12.5) 4.79, d (12.5)	64.1, CH_2_	4.24, d (12.5) 4.33, d (12.5)	10.8, CH_3_	1.85, s
19	176.4, C		176.5, C		177.0, C	
COCH_3_	170.9, C					
COCH_3_	21.1, CH_3_	2.07, s				

^a^ Signals were overlapped.

*Flavuglaucin B* (**7**): amorphous solid; [α]^20^_D_ + 48.9 (*c* 0.09, CH_2_Cl_2_); ^1^H (CDCl_3_, 500 MHz) and ^13 ^C (CDCl_3_, 100 MHz) NMR, see Table 2; HRTOFESIMS *m/z* 337.1399 [M + Na]^+^ (calcd for C_19_H_22_O_4_, 337.1392).

*Flavuglaucin C* (**8**): amorphous solid; [α]^20^_D_ + 38 (*c* 0.05, CH_2_Cl_2_); ^1^H (CDCl_3_, 500 MHz) and ^13 ^C (CDCl_3_, 100 MHz) NMR, see Table 2; HRTOFESIMS *m/z* 315.1593 [M + H]^+^ (calcd for C_19_H_22_O_4_, 315.1596).

*Compound* ***9***: amorphous solid; [α]^20^_D_ + 260.9 (*c* 0.06, CH_2_Cl_2_); spectroscopic data (MS, NMR) identical to those reported in the literature^27^.

### 2.5. Molecular modelling

Flavuglaucin B (**7**) was docked considering the global minimum energy conformation. The ligand was built within the Maestro platform and the most stable conformation has been determined by molecular mechanics conformational analysis performed with Macromodel software version 9.2^28^. In particular the molecule was submitted to a conformational search of 1000 steps with an energy window for saving structure of 21 kJ/mol (5.02 kcal/mol). The algorithm used was the Monte Carlo method followed by energy minimisation carried out using the MMFFs^29^, the GB/SA water implicit solvation model^30^ and the Polak-Ribier Coniugate Gradient (PRCG) method for 5000 iterations, converging on gradient with a threshold of 0.05 kJ/molÅ.

#### 2.5.1. Protein preparation

The coordinates for reverse transcriptase enzyme were taken from the RCSB Protein Data Bank (PDB codes 1RTI)^31^. The protein was prepared by using the Maestro Protein Preparation Wizard. Original water molecules were removed. Also the mutated enzyme A508V-RT was generated starting from wt protein. Mutated RT was minimised considering OPLS^32^ force field in GB/SA^30^ implicit water, setting 10,000 steps interactions analysis with Polak-Ribier Coniugate Gradient (PRCG) method and a convergence criterion of 0.1 kJ/molÅ.

#### 2.5.2. Docking experiments

The docking experiments were performed applying QM-Polarised Ligand Docking (QMPLD)^33^. In order to better take into account the induced fit phenomena, the most energy favoured generated complexes were fully optimised using OPLS^32^ united atoms force field in GB/SA implicit water^30^, setting 10,000 steps interactions analysis with Polak-Ribier Coniugate Gradient (PRCG) method and with a convergence criterion of 0.1 kJ/(molÅ). The resulting complexes were considered for the binding modes graphical analysis with Pymol^34^ and Maestro^35^.

### 2.6. Biochemistry studies

#### 2.6.1. Expression and purification of recombinant HIV-1 RT

HIV-1 RT group M subtype B. Heterodimeric RT was expressed essentially as previously described^13^. Briefly, *E. coli* strain M15 containing the p6HRT-prot vector was grown to an optical density at 600 nm of 0.7 and induced with 1.7 mM isopropyl β-D-1-thiogalactopyranoside (IPTG) for 4 h. Protein purification was carried out with a BioLogic LP system (Biorad), using a combination of immobilised metal affinity and ion exchange chromatography. Cell pellets were resuspended in lysis buffer (50 mM sodium phosphate buffer pH 7.8, containing 0.5 mg/mL lysozyme), incubated on ice for 20 min, and after adding NaCl to a final concentration of 0.3 M, were sonicated and centrifuged at 0.30 × g for 1 h. The supernatant was loaded onto a Ni^2+^-NTA-Sepharose column pre-equilibrated with loading buffer (50 mM sodium phosphate buffer pH 7.8, containing 0.3 M NaCl, 10% glycerol, and 10 mM imidazole) and washed thoroughly with wash buffer (50 mM sodium phosphate buffer pH 6.0, containing 0.3 M NaCl, 10% glycerol, and 80 mM imidazole). RT was eluted with an imidazole gradient in wash buffer (0–0.5 M). Fractions were collected, protein purity was checked by SDS-PAGE and found to be higher than 90%. The 1:1 ration between the p66/p51 subunits was also verified. Enzyme-containing fractions were pooled and diluted 1:1 with 50 mM sodium phosphate buffer pH 7.0, containing 10% glycerol; and then loaded into a Hi-trap heparin HP GE (Healthcare Lifescience) pre-equilibrated with 10 column volumes of loading buffer (50 mM sodium phosphate buffer pH 7.0, containing 10% glycerol and 150 mM NaCl). The column was then washed with loading buffer and the RT was eluted with Elute Buffer 2 (50 mM sodium phosphate pH 7.0, 10% glycerol, 1 M NaCl). Fractions were collected, protein was dialysed and stored in buffer containing 50 mM Tris-HCl pH 7.0, 25 mM NaCl, 1 mM EDTA, and 50% glycerol. Catalytic activities and protein concentrations were determined. Enzyme-containing fractions were pooled and aliquots were stored at −80 °C.

#### 2.6.2. Hiv-1 DNA polymerase-independent RNase H activity determination

HIV RT-associated RNase H activity was measured as described^36^ using the RNase H inhibitor RDS1759^11^ as a control. In 100 µL reaction volume containing 50 mM Tris-HCl buffer pH 7.8, 6 mM MgCl_2_, 1 mM dithiothreitol (DTT), 80 mM KCl, 0.25 µM hybrid RNA/DNA 5′-GAUCUGAGCCUGGGAGCU-Fluorescin-3′ (HPLC, dry, QC: Mass Check) (available from Metabion) 5′-Dabcyl-AGCTCCCAGGCTCAGATC-3′ (HPLC, dry, QC: Mass Check), increasing concentrations of inhibitor, whose dilution were made in water, and 20 ng of wt RT according to a linear range of dose-response curve. The reaction mixture was incubated for 1 h at 37 °C, stopped by addition of EDTA and products were measured with a multilabel counter plate reader Victor 3 (Perkin Elmer model 1420–051) equipped with filters for 490/528 nm (excitation/emission wavelength).

#### 2.6.3. Hiv-1 RNA-dependent DNA polymerase activity determination

RNA-dependent DNA polymerase (RDDP) activity was measured as described^37^ using the NNRTI Efavirenz as a control. In 25 µL volume containing 60 mM Tris-HCl buffer pH 8.1, 8 mM MgCl_2_, 60 mM KCl, 13 mM DTT, 2.5 µM poly (A)- oligo (dT), 100 µM dTTP, increasing concentrations of inhibitor, whose dilution were made in water, and 6 ng of wt RT according to a linear range of dose-response curve. After enzyme addition, the reaction mixture was incubated for 30 min at 37 °C and the stopped by addition of EDTA. Reaction products were detected by picogreen addition and measured with a multilabel counter plate reader Victor 3 (Perkin Elmer model 1420–051) equipped with filters for 502/523 nm (excitation/emission wavelength).

#### 2.6.4. Site-directed mutagenesis

The QuikChange mutagenesis kit (Agilent Technologies Inc., Santa Clara, CA) was used to introduce amino acid substitutions into the p66 HIV-1 RT subunit coded in a p6HRT- prot plasmid by following the manufacturer’s instructions.

## 3. Results and discussion

### 3.1. Isolation and characterisation

The bioactive extract of *T. flavum* subsp. *glaucum* was subjected to vacuum liquid chromatography (VLC) with solvent mixtures of increasing polarity. 45 fractions were obtained that were combined into six main fractions (F1–F6), on the basis of their similarity in TLC. The fractions were further tested in the RT RNase H inhibition assay. The screening showed that the anti-RNase activity was concentrated in three fractions (F1–F3) and, in particular, on the third one which was able to inhibit this function with an IC_50_ of 9.9 µg/mL (Table 1).

**Figure 1.**
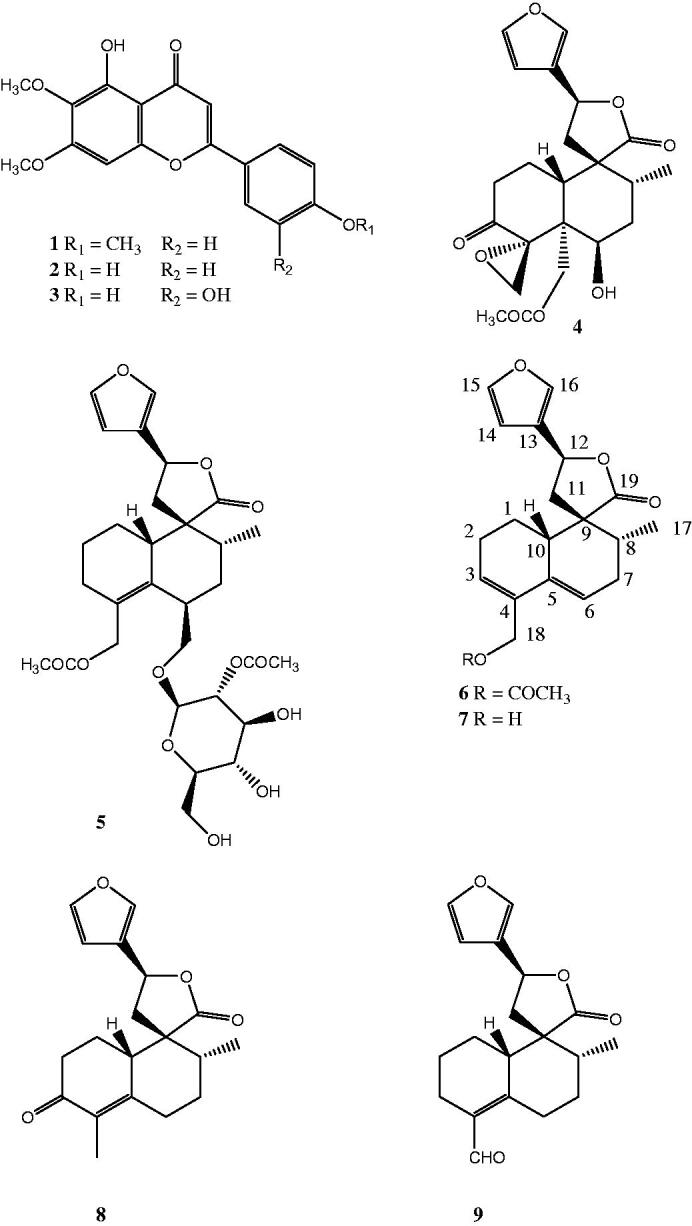
Structures of the isolated and hydrolysed compounds.

Therefore, we decided to purify the three most active fractions using chromatographic techniques such as column chromatography, solid phase extraction (SPE), VLC and semi-preparative HPLC to get three flavones (**1**–**3**) and a *neo*-clerodane, teuflavin (**4**) (Figure 1). With the aim to find a structure-activity relationship (SAR), we also decided to purify the inactive fraction F4 resulting in the isolation of a further *neo*-clerodane, teuflavoside (**5**) (Figure 1). The ^1^H NMR spectra of F5 and F6 revealed that teuflavoside was the main secondary metabolite of these fractions and, as consequence, they were not purified. The structures of the isolated compounds were deduced from the 1 D and 2 D NMR spectra and confirmed by comparison of ^1^H- and ^13 ^C NMR data with those reported in the literature^27^^,^^38–40^.

Given the high percentage of teuflavoside in the extract (13% of the extract), we decided to verify whether the hydrolysis of teuflavoside would lead to an aglycone with inhibitory activity on the RT RNase H function. Therefore, this compound was subjected to acid hydrolysis with 2 N H_2_SO_4_ at reflux for 20 min. TLC analysis of the crude product revealed different spots and, as consequence, the mixture was separated by column chromatography and semi-preparative HPLC (RP HPLC) to give one known (**9**) and three new (**6**–**8**) clerodane diterpenes (Figure 1).

**Figure 2.**
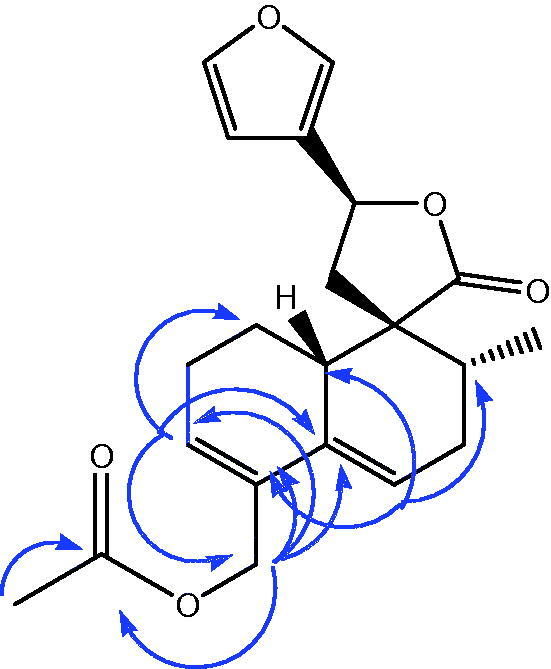
Key HMBC correlations of compound **6**.

Compound **6** showed an ion peak at *m/z* 379.1519 (M + Na) (calcd 379.1515) in the HR-ESIMS (positive mode), accounting for an elemental composition of C_21_H_24_O_5_. The comparison of the ^1^H and ^13 ^C NMR spectra of compound **6** with those of teuflavoside (**5**) showed that the oxymethinic proton at position 6 (δ 4.82, 1H, m) and the 2′-O-acetyl-β-D-glucopyranoside moiety of teuflavoside disappeared in the spectrum of **6** (Table 2). Furthermore, in the ^1^H NMR spectrum of **6** appeared two olefinic protons at 5.86 (1H, s, br) and 5.77 (1H, s, br) ppm that were not present in the spectrum of teuflavoside. These changes suggested that hydrolysis of the glycosidic moiety was accompanied by others structural modifications. From the HSQC spectrum of compound **6** it was possible to assign the respective carbons to each proton. In particular, the two olefinic protons at 5.86 and 5.77 ppm were assigned to the carbons at 128.2 and 121.6 ppm, respectively. The long-range correlations observed in the HMBC spectrum of **6** between the methylene protons at 4.61 (1Ha, d, *J* = 12.5 Hz) and 4.79 (1Hb, d, *J* = 12.5 Hz) ppm with the carbons at δ 170.9 (COCH_3_), 131.6 (C-4), 130.6 (C-5) and 128.2 (C-3), and the olefinic proton at 5.86 with the carbons at 130.6 (C-5), 65.3 (C-18) and 24.2 (C-1) ppm, and the olefinic proton at 5.77 with the carbons at 131.6 (C-4), 45.2 (C-10) 34.6 (C-7) ppm (Figure 2), clearly indicate the presence of two double bonds at position C-3/C-4 and C-5/C-6. ROESY experiments and analysis of scalar (^3^*J*_H-H_) coupling of H-8, H-12 and H-17 confirmed the same stereochemistry of teuflavoside. DQF-COSY, HSQC, HMBC, and ROESY experiments allowed the complete assignment of all signals and the identification of the structure as reported in Figure 1 (Supplementary information, figures S1–S7). It is plausible that the acid hydrolysis of teuflavoside involved the loss of the sugar, dehydration, and subsequent rearrangement of the double bonds (Supplementary information, figure S8). Compound **6** is new to the literature and was trivially named flavuglaucin A.

Compound **7** showed an ion peak at *m/z* 337.1399 (M + Na) (calcd 337.1392) in the HR-ESIMS (positive mode), accounting for an elemental composition of C_19_H_22_O_4_. The ^1^H NMR spectrum of compound **7** (Table 2) was almost superimposable to that of compound **6**, except for the absence of the acetyl group (δ 2.07, s), and the shift of 0.4 ppm to high fields of the oxymethylene protons located at C-18. The absence of the acetyl group in **8** was confirmed by HMBC experiments highlighting no cross-peaks between the oxymethylene protons at C-18 (H-a: 4.24 (d, 1H, *J* = 12.5 Hz); H-b: 4.33 (d, 1H, *J* = 12.5 Hz)) and carbonyl groups. As far as we know, compound **7** is new to the literature and was trivially named flavuglaucin B.

**Figure 3.**
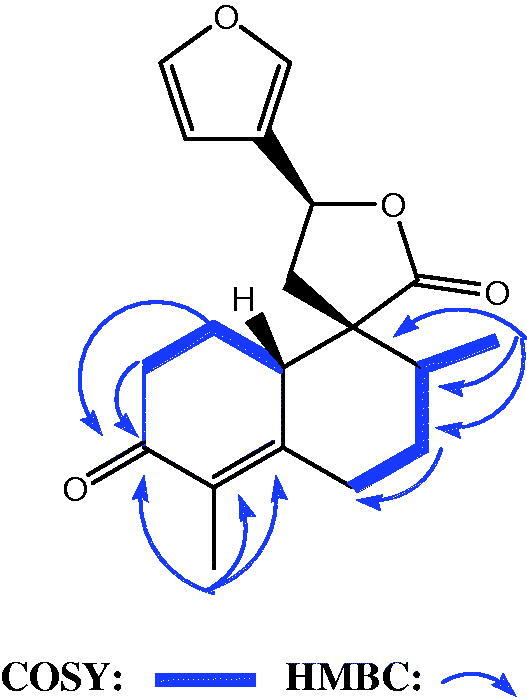
Selected COSY and HMBC correlations of compound **8**.

The HR-ESIMS of compound **8** showed a molecular ion at *m/z* 315.1593 (M + H)^+^ (calcd 315.1596). This molecular mass in combination with ^1^H and ^13 ^C NMR data allowed the molecular formula to be established as C_19_H_22_O_4_. The analysis of the ^1^H NMR spectrum of compound **8** showed that the low field region is similar to that of teuflavoside but the olefinic protons at C-3/C-4 and C-5/C-6 of compounds **6** and **7**, are absent in **8** (Table 2). Moreover, a methyl group at 1.85 ppm (3H, s) appeared in the spectrum of **8**. The HMBC spectrum of **8** revealed that the above mentioned methyl group was correlated with a carbonyl at 197.5 ppm and two unsaturated quaternary carbons at 131.7 and 155.0 ppm (Figure 3). In the same spectrum, the carbonyl group at 197.5 was correlated to the methylene protons at 1.89 (1H-a, m) and 2.31 (1H-b, m). In the HMBC spectrum of **8**, further correlations of methyl group at 1.03 (1H, d, *J* = 6.5 Hz) ppm with carbons at 38.9, 54.5 and 29.1 ppm together with those of the methylene proton at 1.72 (dd, *J* = 14, 3.5) with the methylene at 2.05 (dd, *J =* 13.5, 3.5) ppm observed in the COSY spectrum, allowed to identify the structure of the decalinic nucleus (Figure 3). DQF-COSY, HSQC, HMBC, and ROESY experiments allowed the complete assignment of all signals and the identification of the structure as reported in Figure 1. Compound **8** is a previously undescribed molecule and was trivially named flavuglaucin C.

The structure of compound **9** (Figure 1) was deduced from the study of 1 D and 2 D NMR spectra and MS and confirmed by comparison with the spectral data reported in the literature^27^.

### 3.2. Inhibitory effects on HIV-1 RT-associated functions and structure-activity relationships

The compounds isolated from the active fractions of *T. flavum* extract were evaluated for their anti-RNase H activity (Table 3) using as positive control RDS1759, a diketoacid inhibitor of the RNase H function that binds the catalytic site^11^. The assays revealed that the anti-RNase H activity of the extract was mainly due to the flavone cirsiliol (**3**) with an IC_50_ of 8.2 µM and to a much lower extent (IC_50_ = 89 µM) to the flavone cirsimaritin (**2**), while the flavone salvigenin completely inactive up to the concentration of 100 µM. The SAR analysis of the three flavones pointed out the importance of the catechol group to inhibit the RNase H function. Indeed, removing the hydroxyl group from C-3′ position of cirsiliol (**3**) lead to cirsimaritin (**2**) with a decrease in the activity of 10 folds. In addition, methoxylation of hydroxy group at C-4′ of cirsimaritin lead to salvigenin (**1**) that was completely inactive (IC_50_ >100 µM). Therefore, the maximum activity occurred when both hydroxyl groups are present at C-3′ and C-4′ of the B ring. The importance of the cathecol group for the inhibition of RNase H function was already observed in our previous work, comparing a series of caffeic and ferulic acid derivatives^25^. The natural *neo*-clerodanes teuflavin (**4**) and teuflavoside (**5**) resulted inactive up to the concentration of 100 µM. The lack of activity of teuflavoside was not surprising because it was purified from an inactive fraction.

**Table 3.** Effect of the isolated and hydrolysed compounds on the HIV-1 RT-associated RNase H and RDDP functions

Compounds	RnaseH IC_50_ (µM)^a^	RDDP IC_50_ (µM)^a^
**1**	>100 (80 %)^b^	ND^c^
**2**	89 ± 7	ND
**3**	8.2 ± 0.6	ND
**4**	>100 (80 %)	ND
**5**	>100 (74 %)	ND
**6**	20.2 ± 2	>100 (80 %)
**7**	9.1 ± 0.2	>100 (80 %)
**8**	52.4 ± 0.4	>100 (80 %)
**9**	36.4 ± 0.4	>100 (80 %)

^a^ Compound concentration required to reduce the enzyme activity by 50%.

^b^ Percentage of residual enzyme activity in the presence of 100 µM of the compound.

^c^ ND, not done.

Among the semi-synthetic *neo*-clerodanes, flavuglaucin B (**7**) showed the greatest inhibitory activity on RNase H function with an IC_50_ of 9.1 µM. Flavuglaucin A (**6**) was about two folds less active when compared with **7**. This data seemed to indicate that the alcohol function was important for interaction with the binding site of the RT-associated RNase H function. Flavuglaucin C (**8**) and compound **9**, containing a methyl or aldehyde group at C-4 position, were about six to four folds less active than flavuglaucin B, respectively. This data confirmed the relevance of the alcoholic function to activity. However, the presence of only one double bond in the decalinic nucleus of compounds **8** and **9** change the molecular planarity and thus could further influence the interaction with RT-associated RNase H function. The *neo*-clerodanes **6–9** were also evaluated against the RT polymerase function (RDDP) but no inhibitory activity was observed up to the concentration of 100 µM (Table 3).

### 3.3. Site-directed mutagenesis experiments

**Figure 4.**
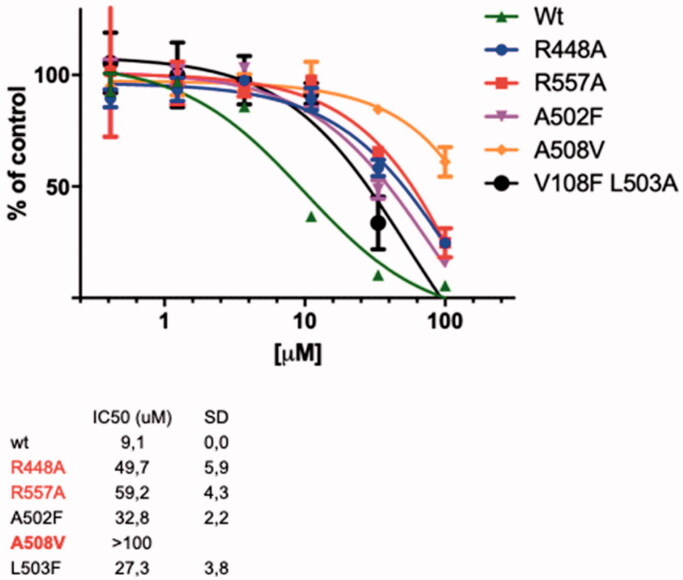
Effect of flavuglaucin B (**7**) on the HIV-1 RT associated RNase H function of mutated RTs.

Since the *neo*-clerodane flavuglaucin B was not able to inhibit the RDDP function and apparently it does not contain any functionality able to bind to the RNase H active site coordinating the Mg^2+^ cofactors, we supposed that this compound might bind an allosteric RT site. In order to verify this hypothesis, it was chosen to perform site-directed mutagenesis, determining the independent impact of several amino acid substitutions on the potency of the compound to inhibit the RNase H function. All the selected aminoacids are localised in the RNase H domain and are potentially crucial for the binding of RNase H function inhibitors. To verify a possible interaction for flavuglaucin B in the allosteric site described by Himmel et al.^16^, residue V108 was replaced by a phenylalanine in order to reduce the binding available space for the compound. Results showed a slight increase in IC_50_ when flavuglaucin B was assessed against V108F, compared with the wild type enzyme (Figure 4).

The next mutation involved the residue A502 located in the alpha helix, close to the second identified allosteric binding pocket. This pocket is located in the RNase H domain, between the RNase H active site and the primer grip region, close to the interface of subunits p66 and p51. A502 residue was replaced by a phenylalanine with the aim to provoke a shift of alpha helix that might reduce the space between the two subunits p51 and p66 and therefore hinder the entrance of the compound in the pocket. Also in this case, flavuglaucin B showed a moderate loss in potency (3.5-fold). Conversely, flavuglaucin B showed a significant loss in potency in the case of R448A (5.5-fold), R557A (6.5-fold) and, especially, A508V that totally impaired the RNase H inhibition by flavuglaucin B (IC_50_ >100 µM). All together these data suggested that flavuglaucin B established strong interactions within the allosteric pocket located between the RNase H active site and the primer grip region, close to the interface of subunits p66 and p51, previously investigated for other allosteric RNase H inhibitors^20^.

### 3.4. Docking experiments

To further investigate the mechanism of action of flavuglaucin B (**7**), we carried out QM polarised ligand (QMPL) docking experiments^33^. The same docking protocol was applied successfully in previous studies^20^^,^^41^. QMPL docking workflow combines docking with ab initio methods for ligand charges calculation within the protein environment. Subsequently, the best poses were subjected to molecular energy minimisation to consider induced-fit protein conformation change (that takes place after ligand binding) and implicit water solvation.

**Figure 5.**
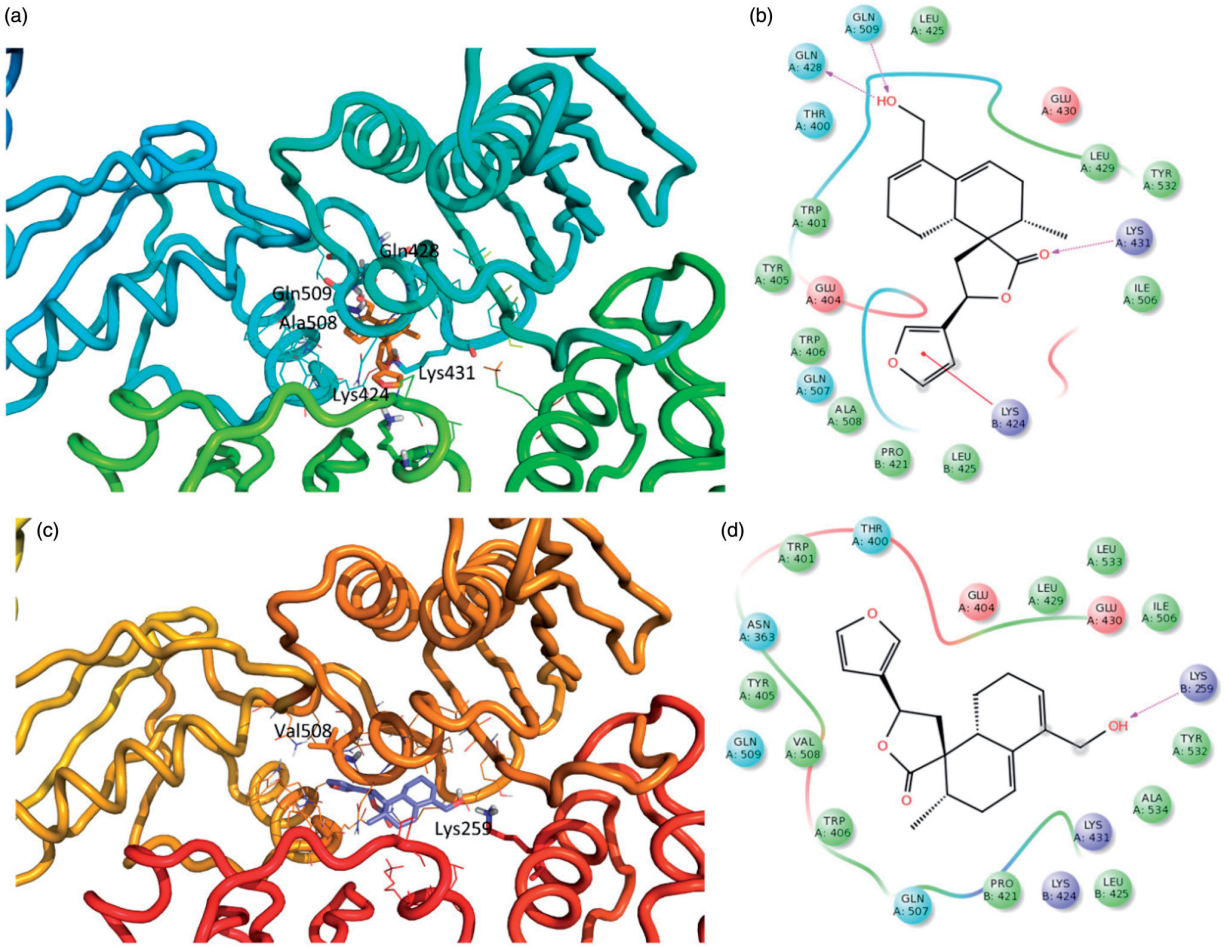
3D representation of the putative binding mode obtained by docking experiments. a) RTwt- flavuglaucin B (**7**) c) A508V-RT- flavuglaucin B (**7**) complex and the relative 2D representation of the complexes stabilising interactions with the binding site residues represented with different colour depending on their chemical-physical properties: green, hydrophobic; cyan, polar; violet, positive; red, negative charged residues. Instead, magenta arrows indicate the formation of hydrogen bond between protein and ligand.

In agreement with site mutagenesis results, these studies suggested that flavuglaucin B binds into an allosteric pocket close to the RNase H catalytic site interacting with several residues through hydrogen bonds: Gln428, Gln509, Lys431, a cation-π with Lys424 and several hydrophobic interactions (e.g. Leu425, Leu429, Tyr532, Ala508) (Figure 5(a,b)). Hence, when bound to this site, flavuglaucin B might induce the RNase H domain to a position in which the active site might no longer be able to catalyse hydrolysis cleavage of the RNA strand in the of RNA: DNA duplex. The single point mutation of residue Ala508 to Val, in an attempt to reduce the space available for flavuglaucin B accommodation, seems to confirm this mechanism of action. The docking results into the mutated enzyme show that the compound is not able to be accommodated in the same position and it loses several important interactions (Figure 5(c,d)).

## 4. Conclusions

A bioguided fractionation of the *T. flavum* subsp*. glaucum* extract permitted to identify the flavone cirsiliol as the main responsible of the inhibitory activity of the RT-associated RNase H function of the extract. As far as we know, the inhibition of the HIV-1 RT-associated RNase H function by cirsiliol has not been reported in the literature. It is interesting to note that cirsiliol was also able to inhibit the HIV-1 integrase at a concentration of 12 µM^42^, suggesting cirsiliol as a dual inhibitor of HIV-1.

As regards the products obtained from the hydrolysis of teuflavoside, detailed NMR studies showed that the acid environment did not lead to the expected aglycone, but a series of clerodanes resulting from dehydration from position 6 and subsequent molecular rearrangement. The results seem to be in agreement with those of Savona et al.^27^ reporting that acid hydrolysis of the 18,2′-bis-deacetylteuflavoside did not lead to the corresponding aglycone. All semi-synthetic compounds (**6–9**) showed inhibitory activity on the RNase H activity and, in particular, the *neo*-clerodane flavuglaucin B was the most potent, with an IC_50_ of 9.1 µM. None of the molecules was able to inhibit the reverse transcriptase RDDP function up to a concentration of 100 µM. To the best of our knowledge, this is the first time that clerodane diterpenes have been identified as inhibitors of HIV-1 RT. Site-directed mutagenesis studies suggested that flavuglaucin B bind to the RT allosteric pocket located between the RNase H active site and the primer grip region, close to the interface of subunits p66 and p51. These results prompt us to undergo further studies to evaluate the activity of the best performing compounds on infected cells and to develop *neo*-clerodane derivatives with more potent anti-RT activity

## Supplementary Material

Supplemental Material
