# Supplementary material for: Flavonoids and Acid-Hydrolysis derivatives of Neo-Clerodane diterpenes from Teucrium flavum subsp. glaucum as inhibitors of the HIV-1 reverse transcriptase–associated RNase H function
Source: J Enzyme Inhib Med Chem. 2021 Mar 10;36(1):749–57. doi: 10.1080/14756366.2021.1887170 (PMC7952052; doi:10.1080/14756366.2021.1887170)
Supplement: Supplemental Material [file IENZ_A_1887170_SM9462.pdf]

Supplementary Material

Flavonoids and Acid-Hydrolysis Derivatives of Neo-Clerodane Diterpenes from *Teucrium flavum* subsp. *glaucum* as Inhibitors of the HIV-1 Reverse Transcriptase–Associated RNase H Function

Benedetta Fois[a], Angela Corona[a], Enzo Tramontano[a,b], Simona Distinto[a], Elias Maccioni[a], Rita Meleddu[a], Pierluigi Caboni[a], Costantino Floris[c], and Filippo Cottiglia[a]*

[a]*Department of Life and Environmental Sciences, University of Cagliari,* Cittadella Universitaria di Monserrato, 09042 Monserrato, Italy

[b]*Istituto di Ricerca Genetica e Biomedica, Consiglio Nazionale delle Ricerche, Monserrato, Italy*

[c]*Department of Chemical and Geological Sciences, University of Cagliari, 09042 Monserrato, Italy*

*Corresponding author. Tel +39-0706758979; fax +39-0706758551; e-mail: cottiglf@unica.it

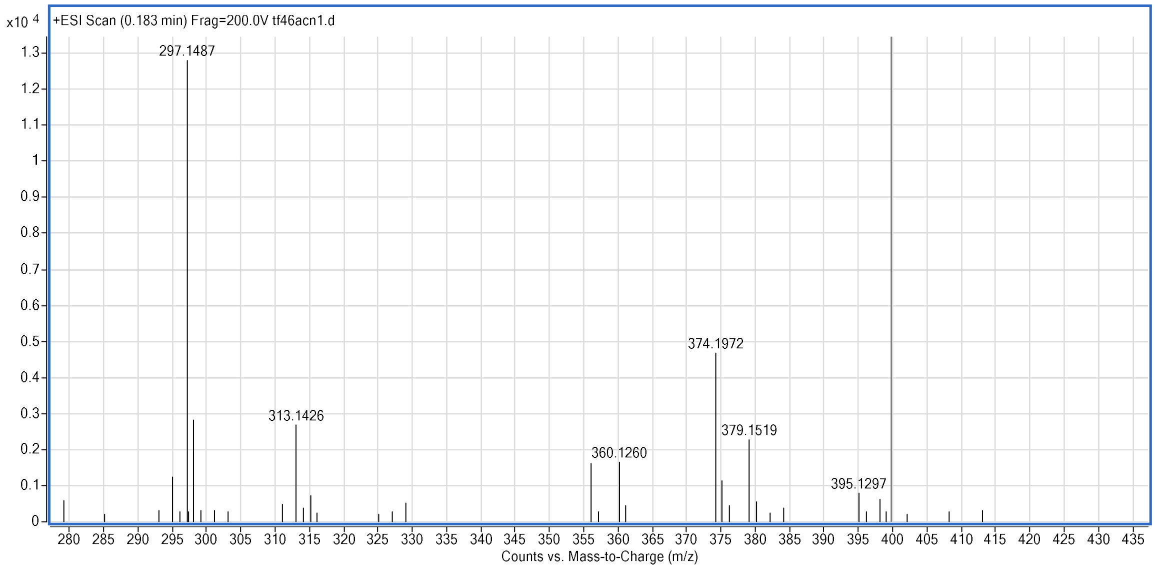

Figure S1: HR ESIMS spectrum (positive mode) of flavuglaucin A (**6**)

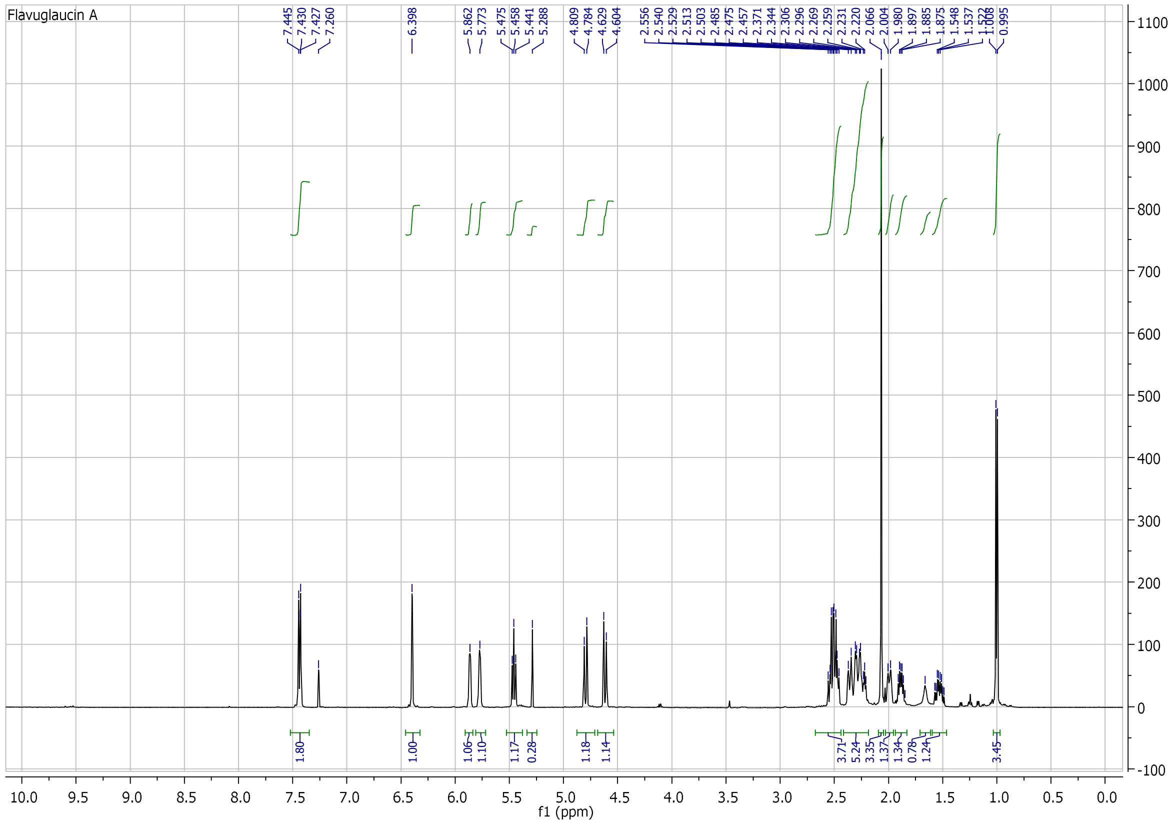

Figure S2: ^{1}H NMR spectrum (500 MHz, $CDCl_3$) of flavuglaucin A (**6**)

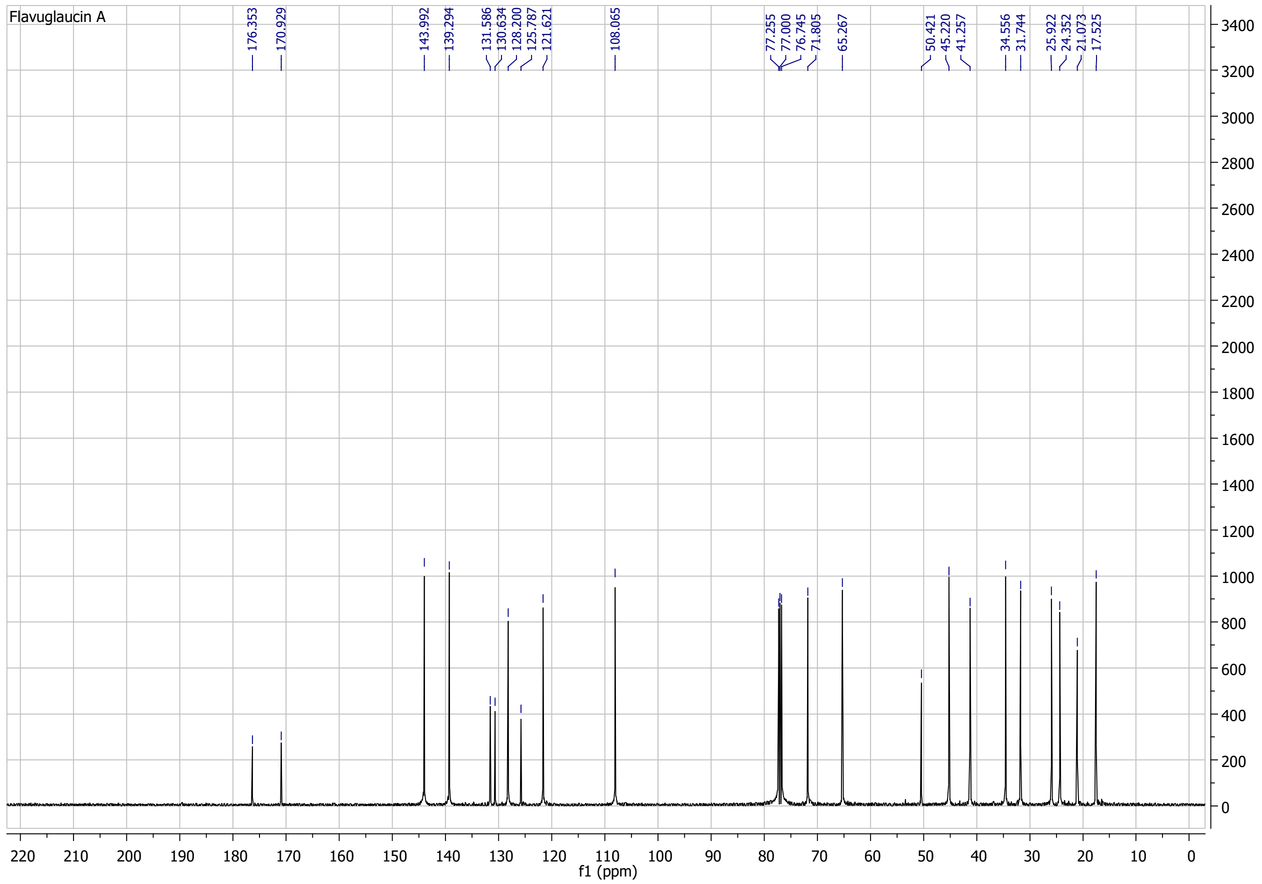

Figure S3: ^{13}C NMR spectrum (100 MHz, $CDCl_3$) of flavuglaucin A (**6**)

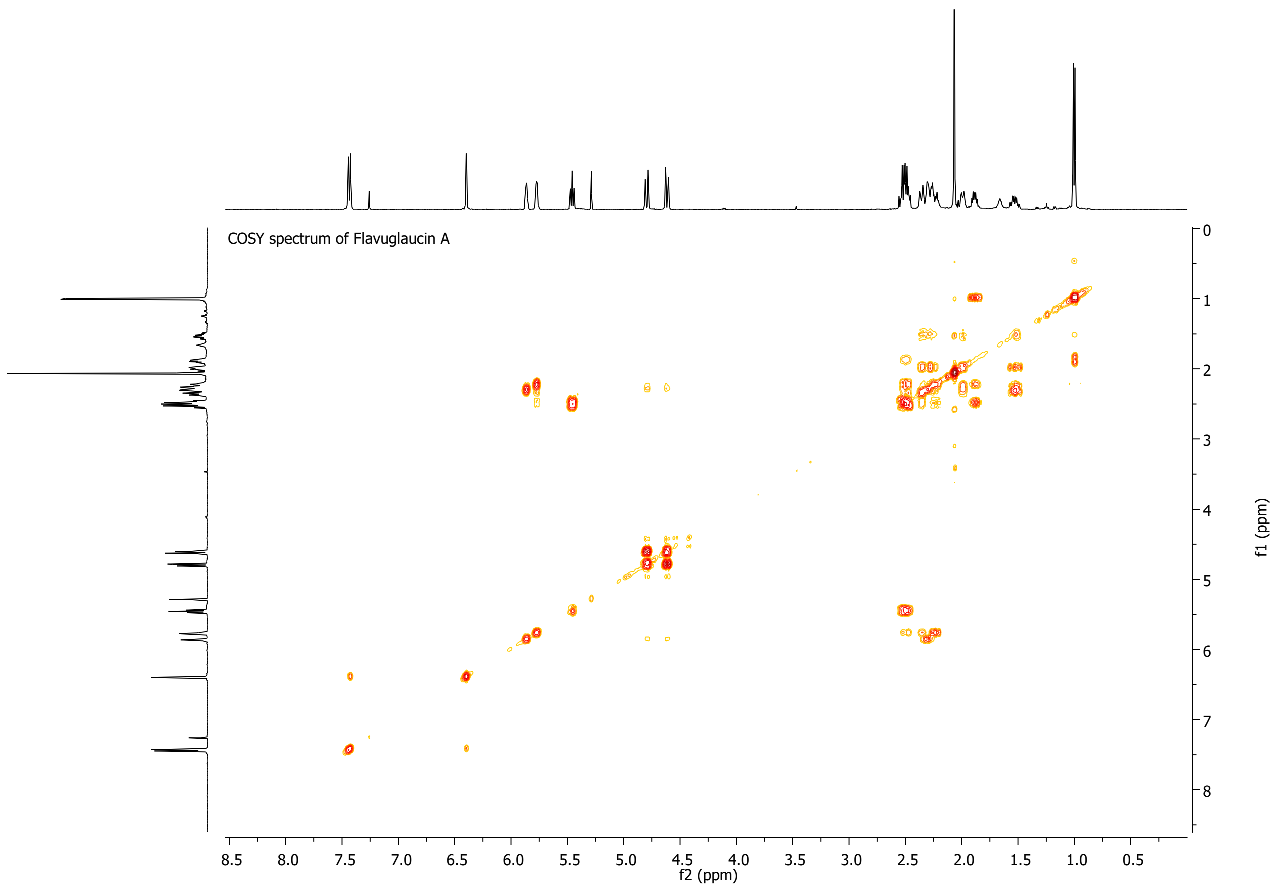

Figure S4: COSY spectrum (500 MHz $CDCl_3$) of flavuglaucin A (**6**)

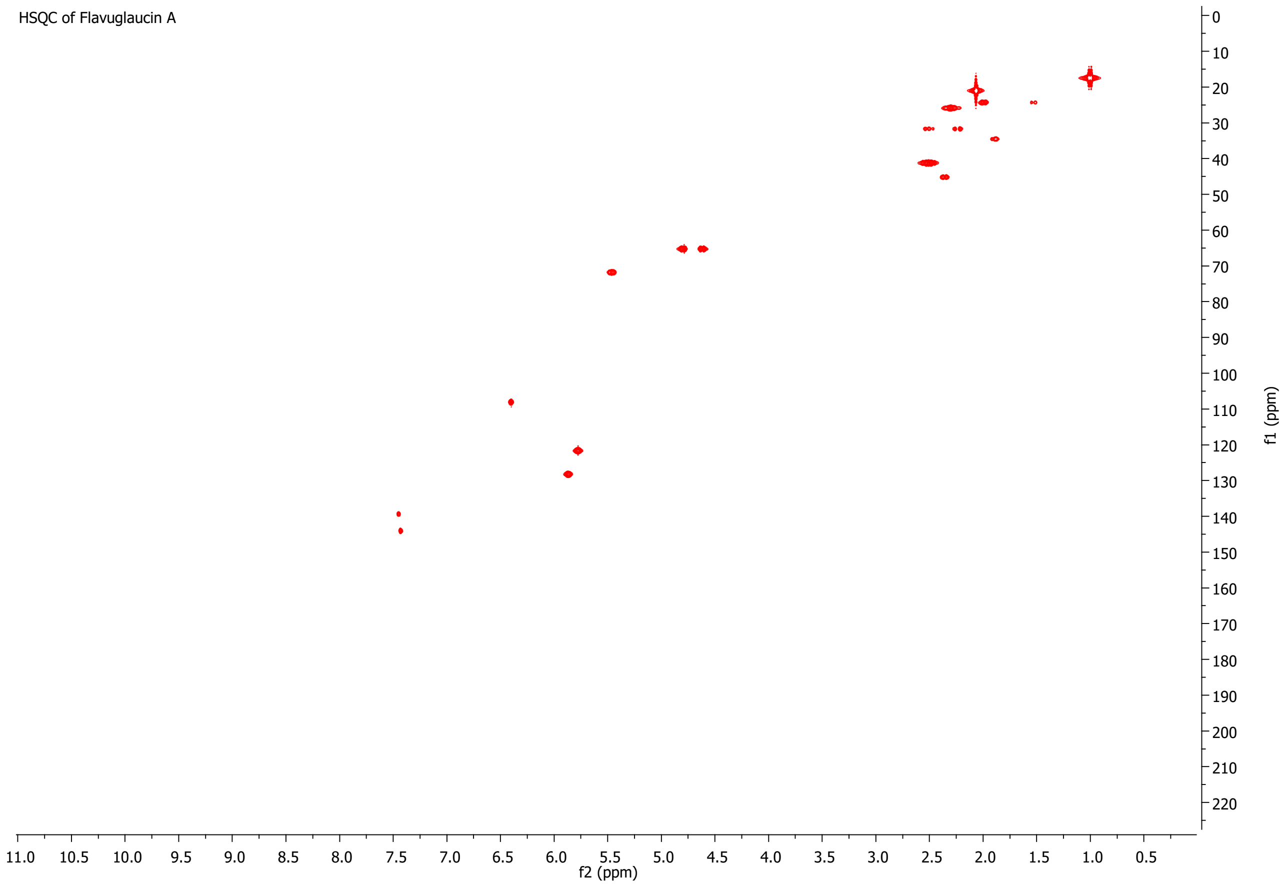

Figure S5: HSQC spectrum (500 MHz $CDCl_3$) of flavuglaucin A (**6**)

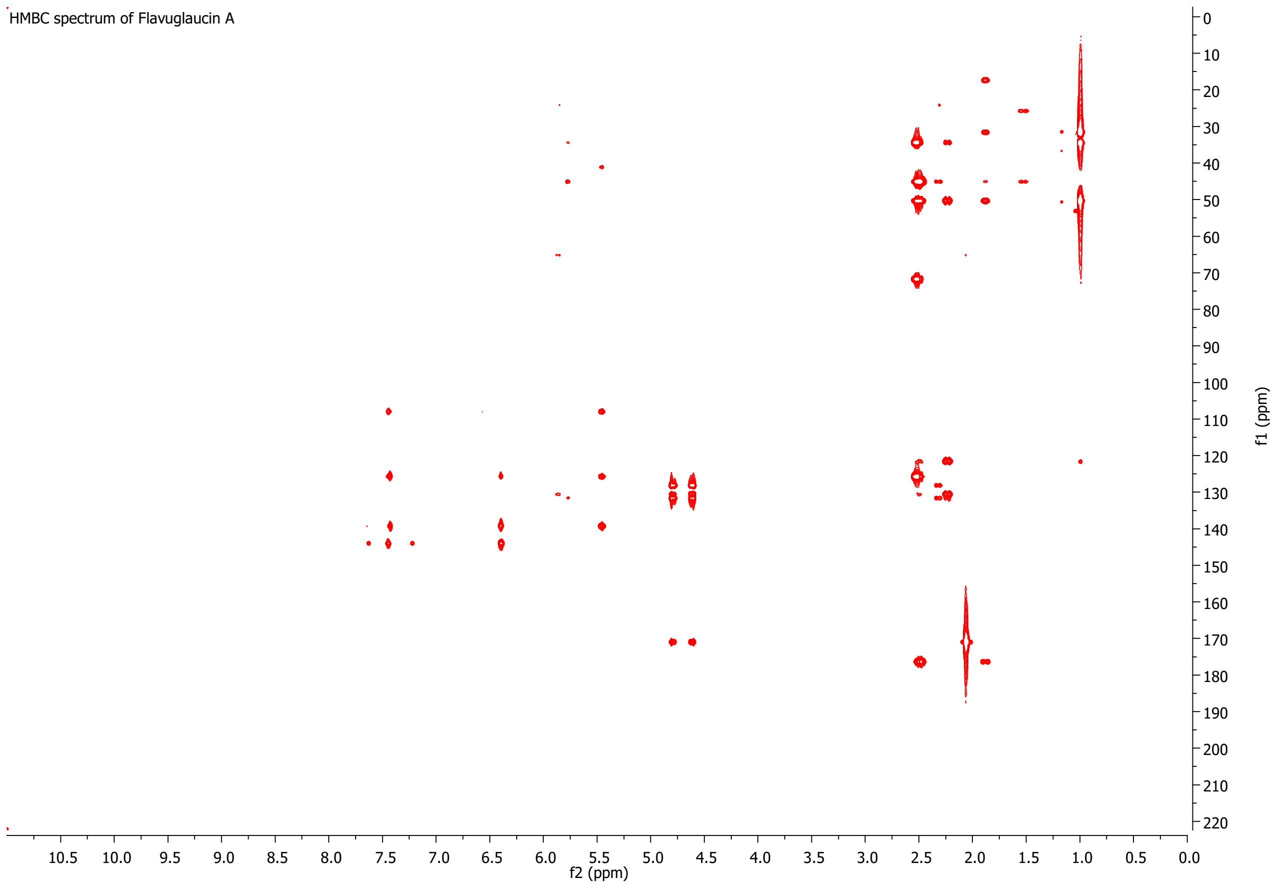

Figure S6: HMBC spectrum (500 MHz $CDCl_3$) of flavuglaucin A (**6**)

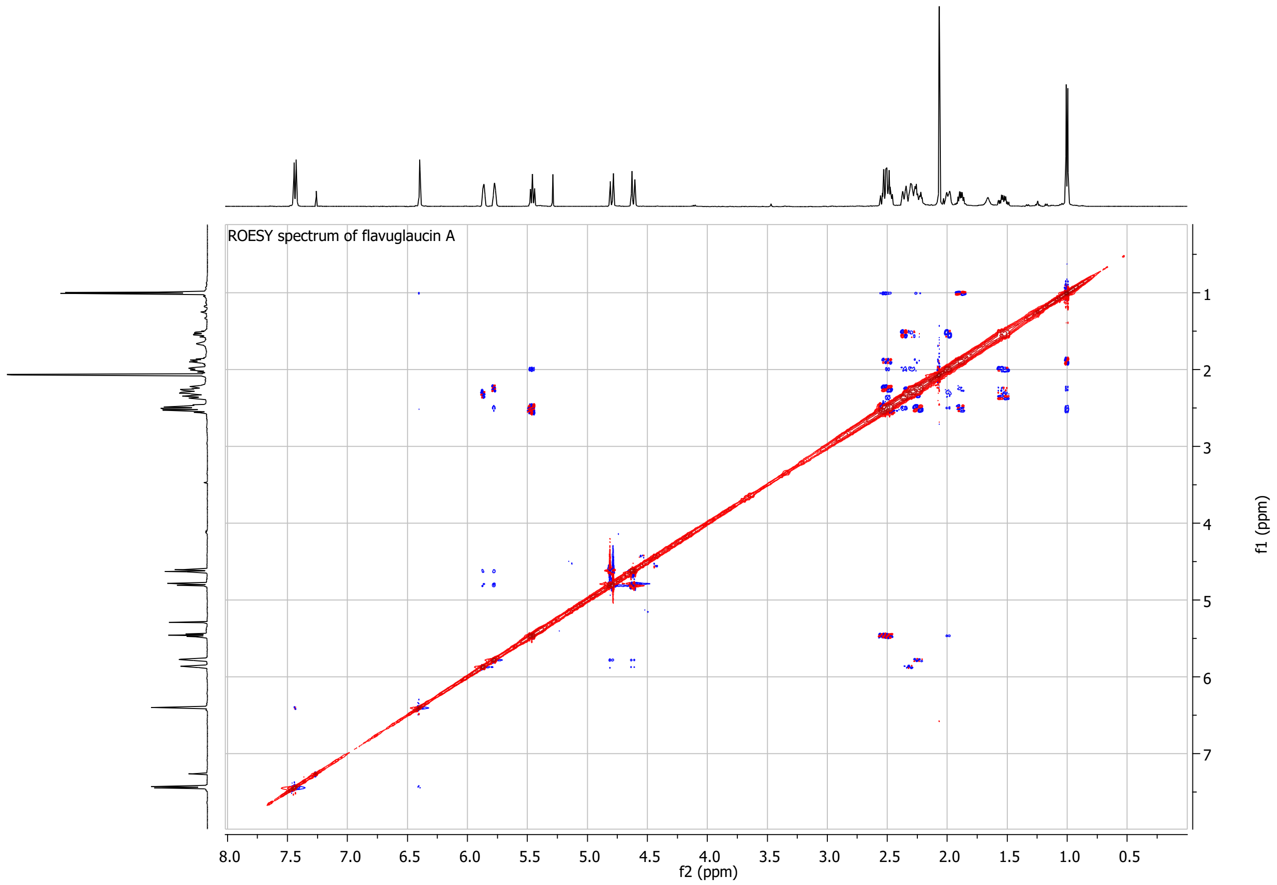

Figure S7: ROESY spectrum (500 MHz, $CDCl_3$) of flavuglaucin A (**6**)

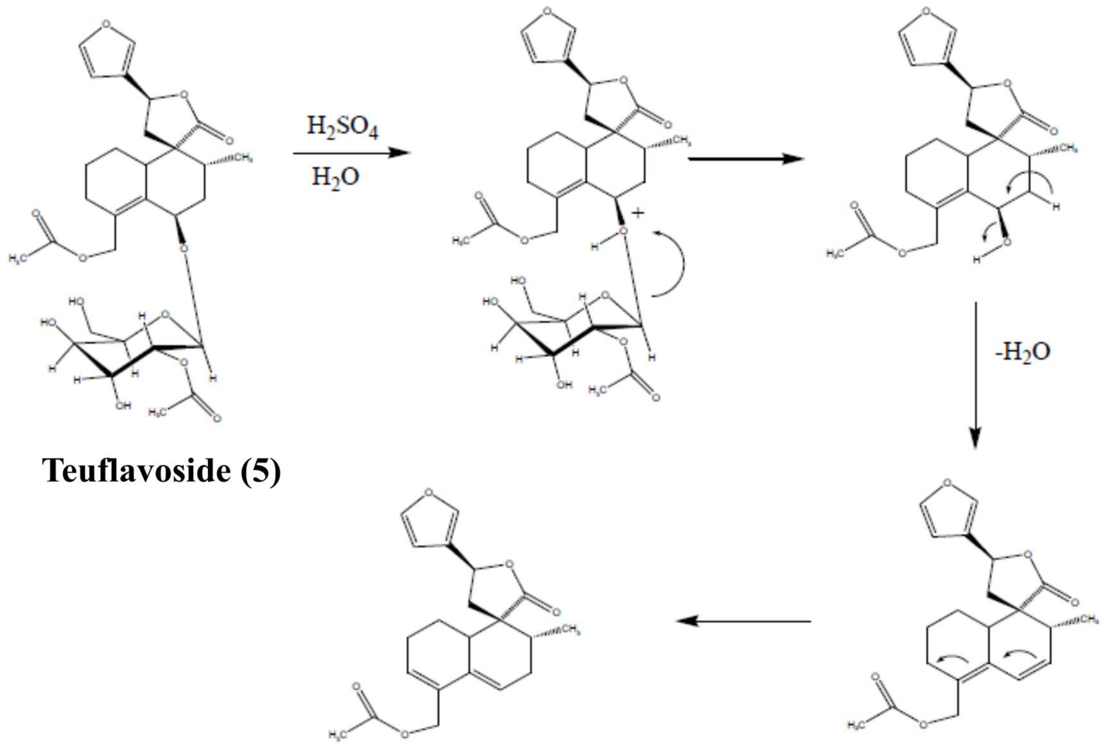

Teuflavoside (5)

Flavuglaucin A (6)

Figure S8: Mechanism of hydrolysis for compound **6**

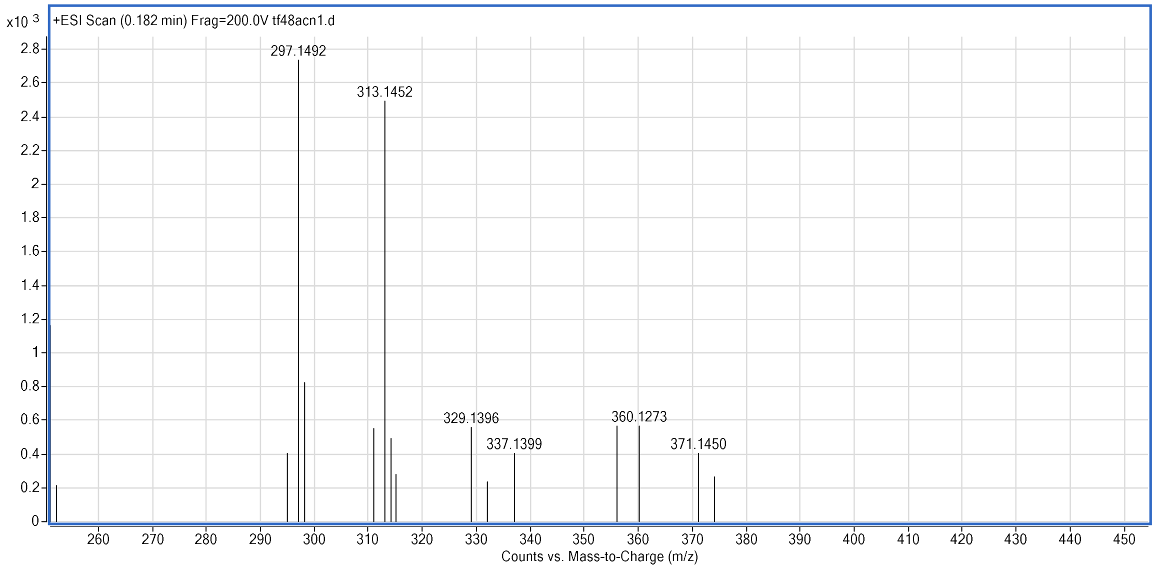

Figure S9: HR ESIMS spectrum (positive mode) of flavuglaucin B (**7**)

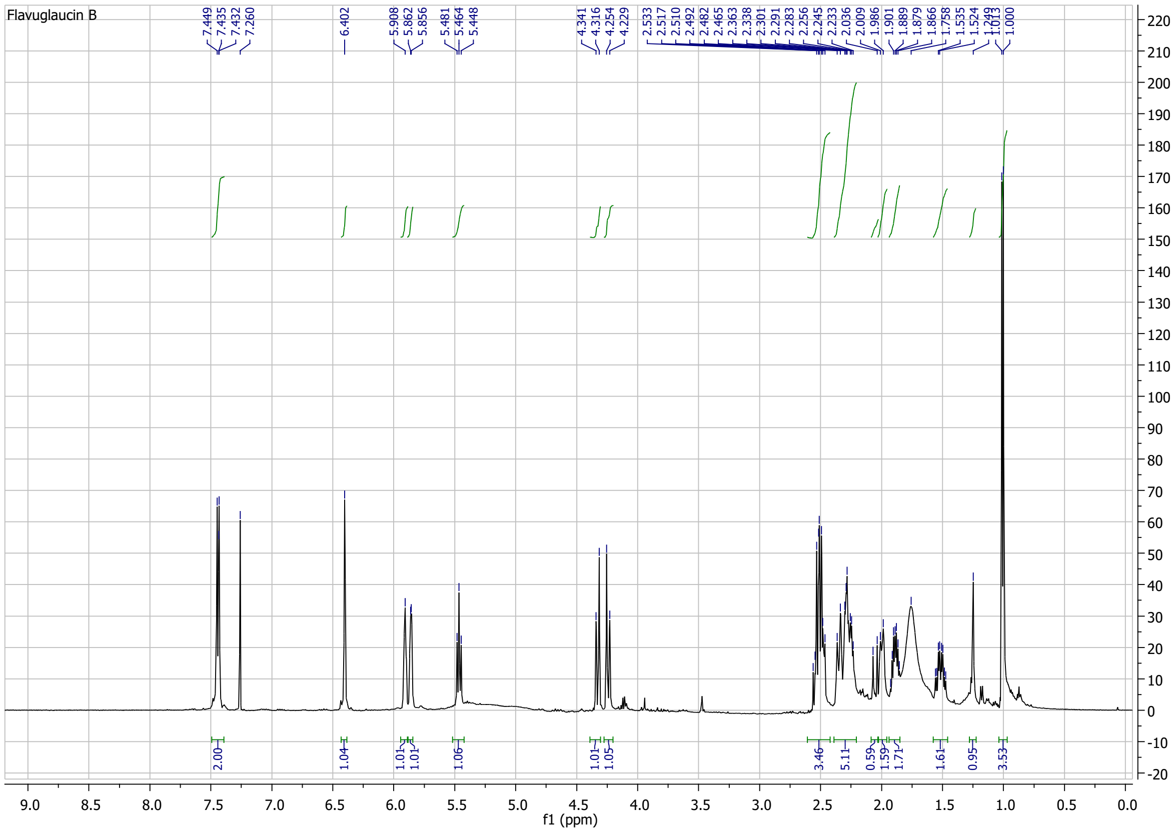

Figure S10: ^{1}H NMR spectrum (500 MHz, $CDCl_3$) of flavuglaucin B (**7**)

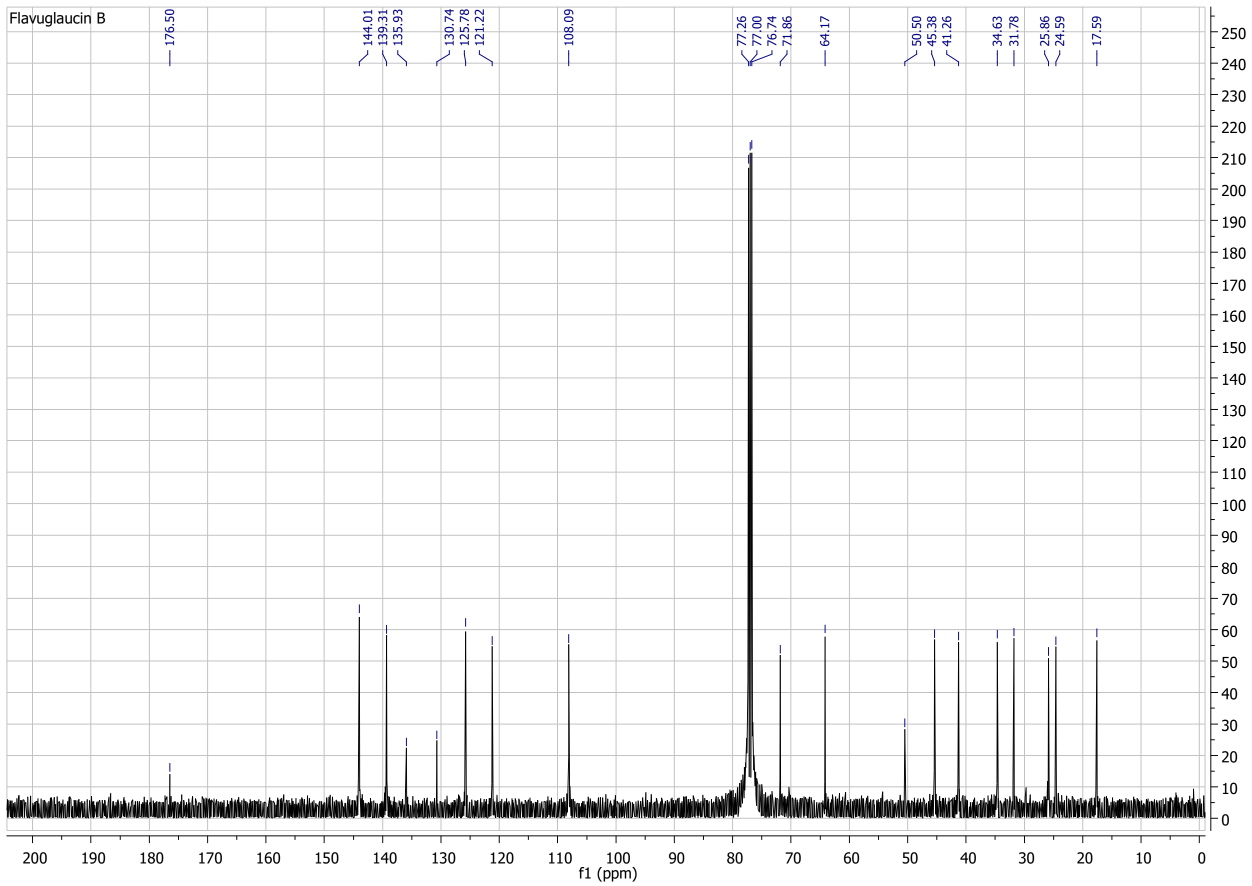

Figure S11: ^{13}C NMR spectrum (100 MHz, $CDCl_3$) of flavuglaucin B (**7**)

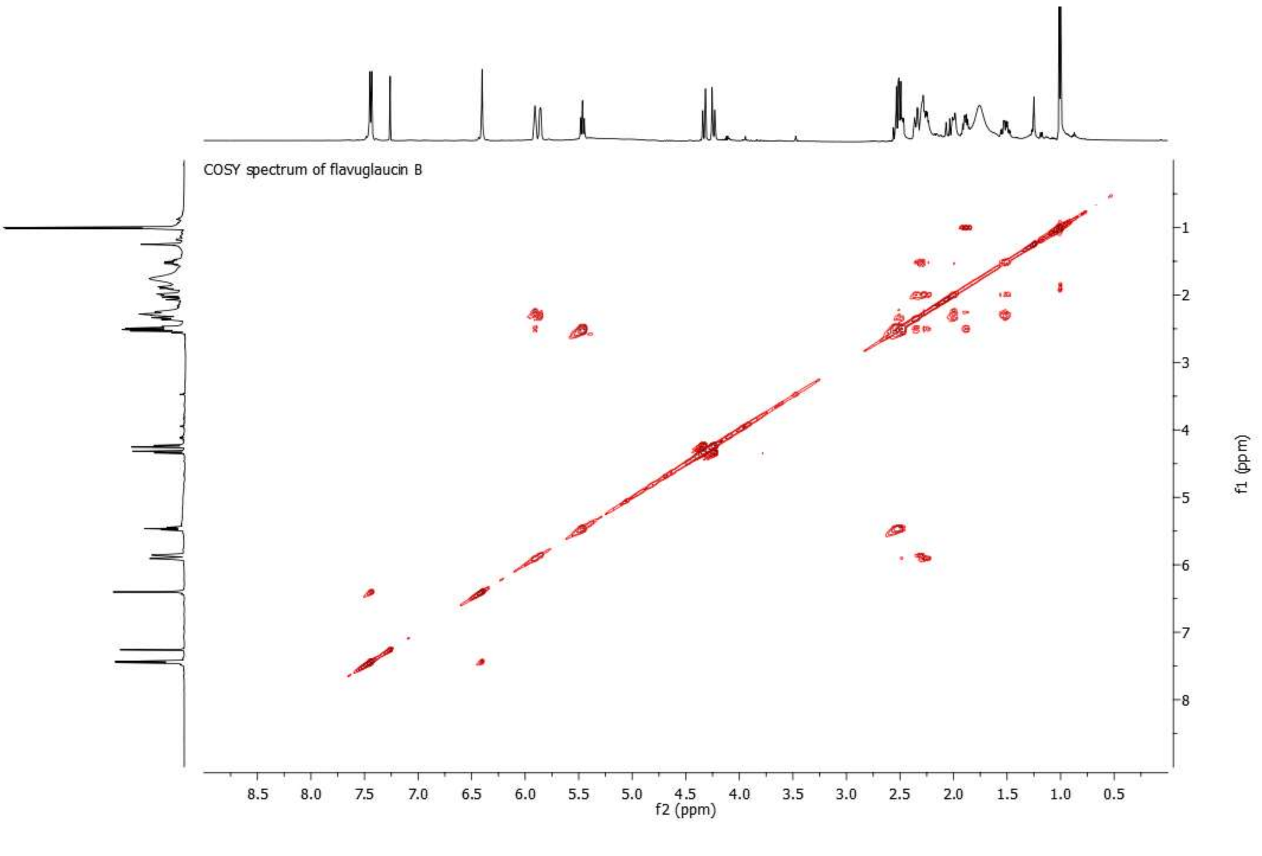

Figure S12: COSY spectrum (500 MHz $CDCl_3$) of flavuglaucin B (**7**)

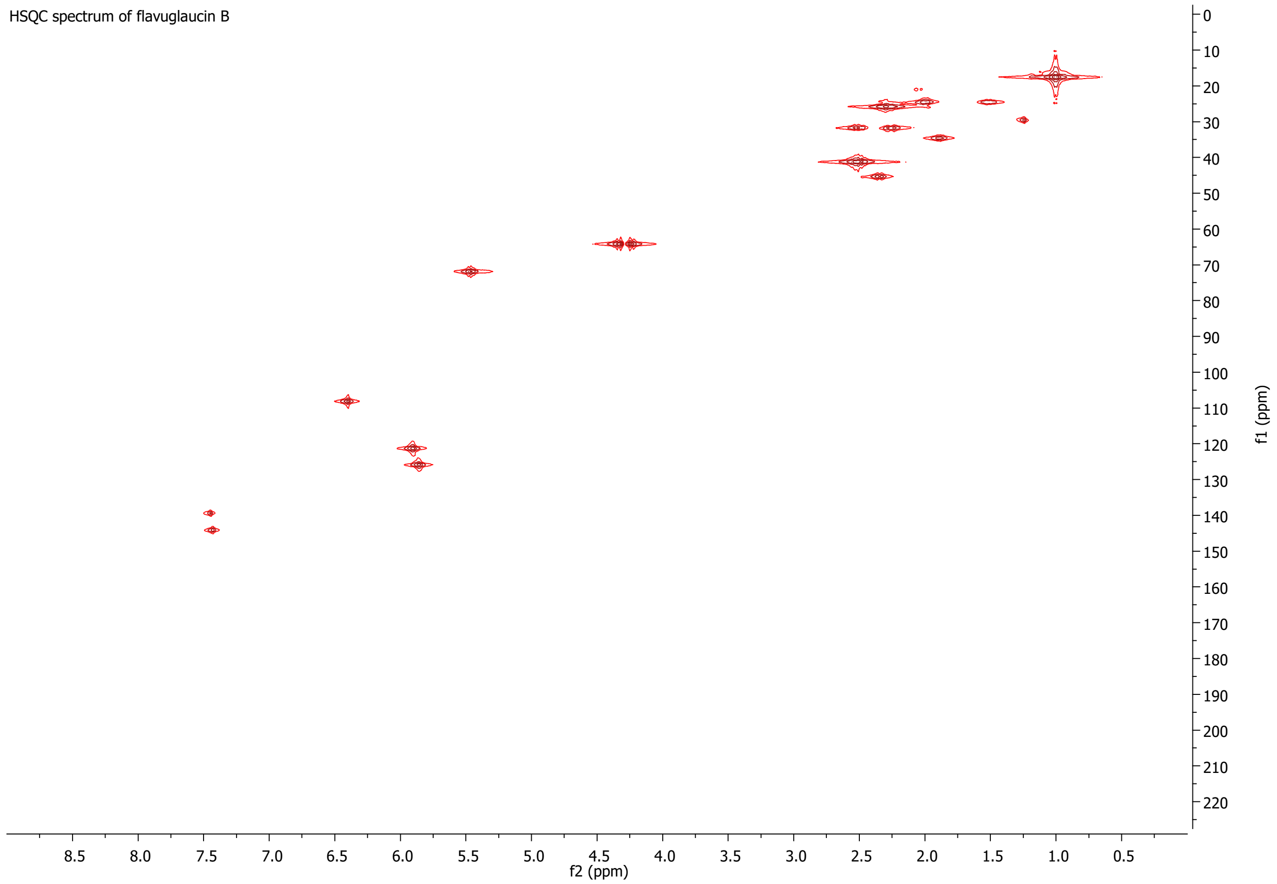

Figure S13: HSQC spectrum (500 MHz $CDCl_3$) of flavuglaucin B (**7**)

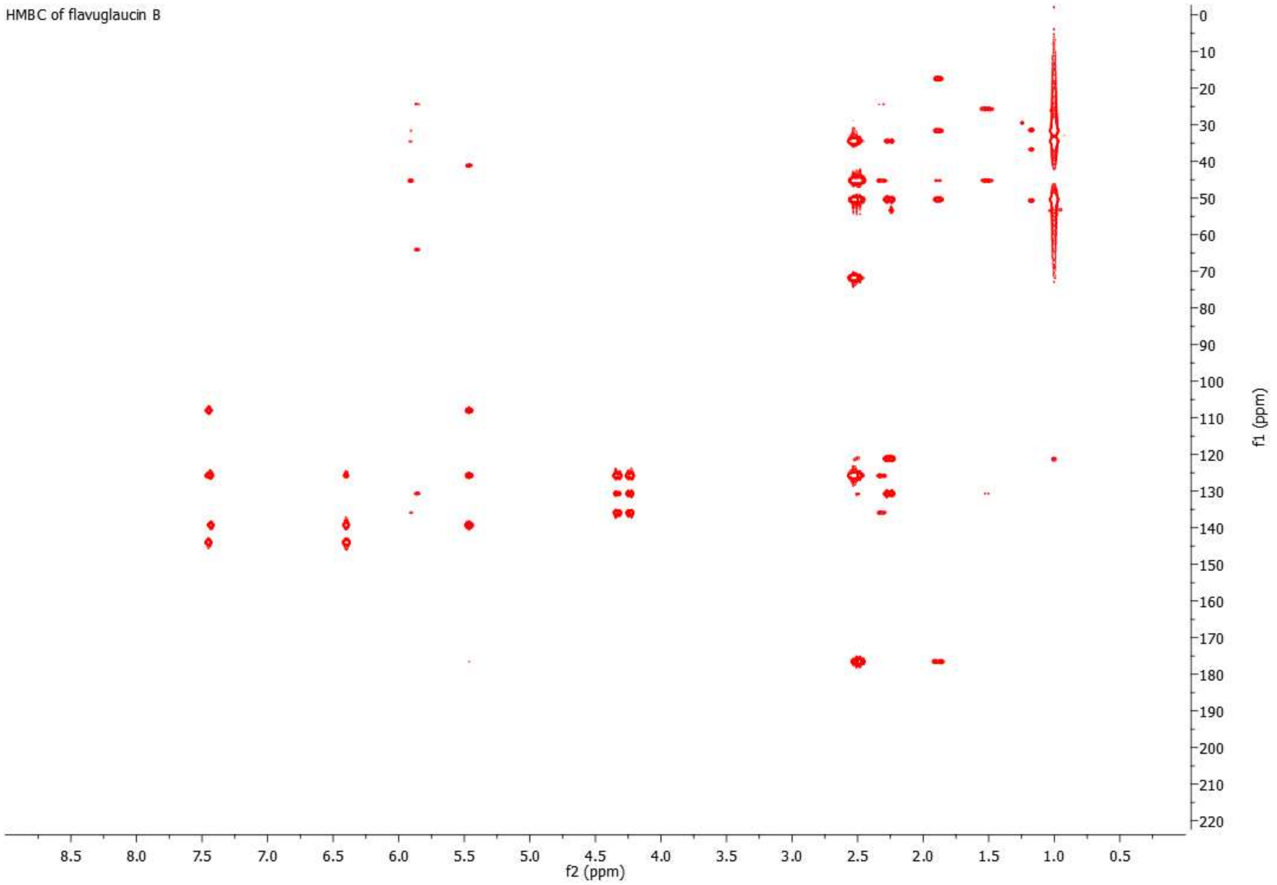

Figure S14: HMBC spectrum (500 MHz $CDCl_3$) of flavuglaucin B (**7**)

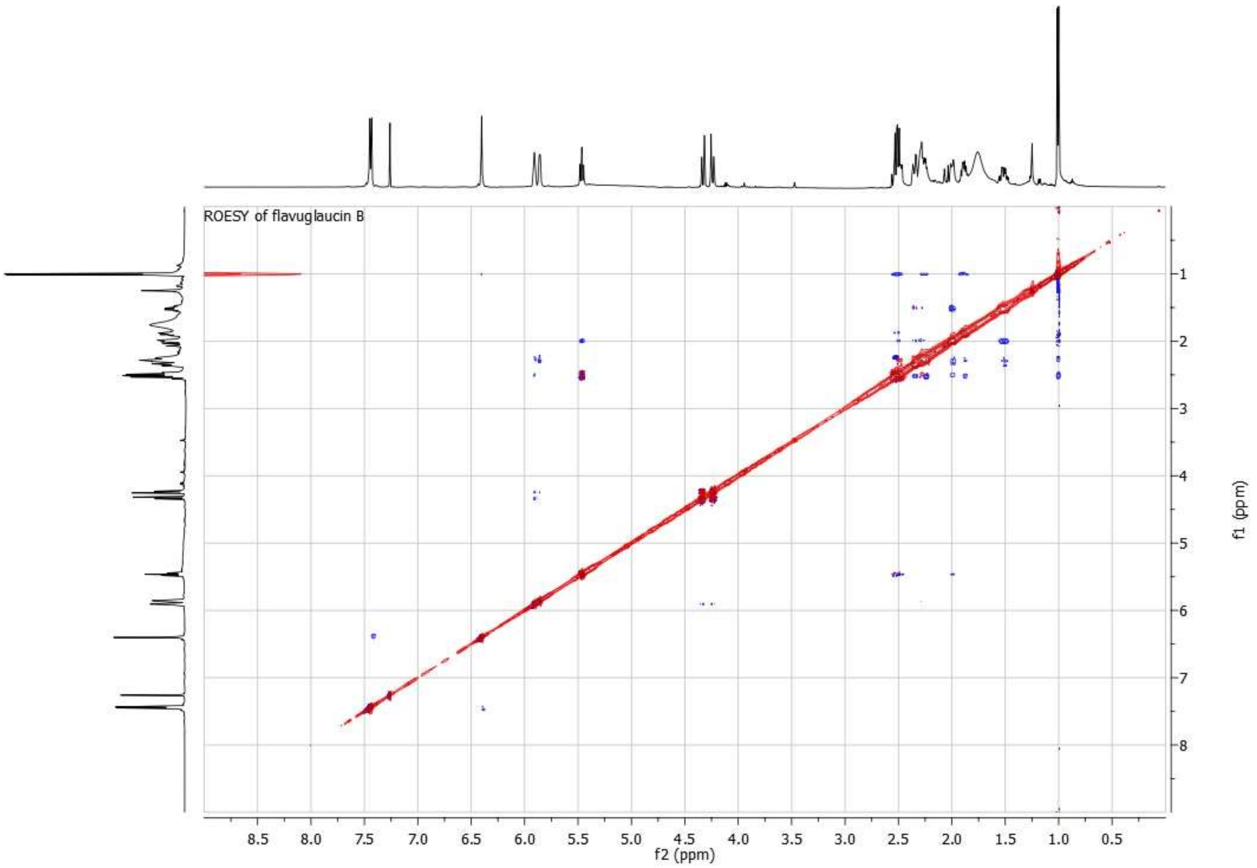

Figure S15: ROESY spectrum (500 MHz, $CDCl_3$) of flavuglaucin B (**7**)

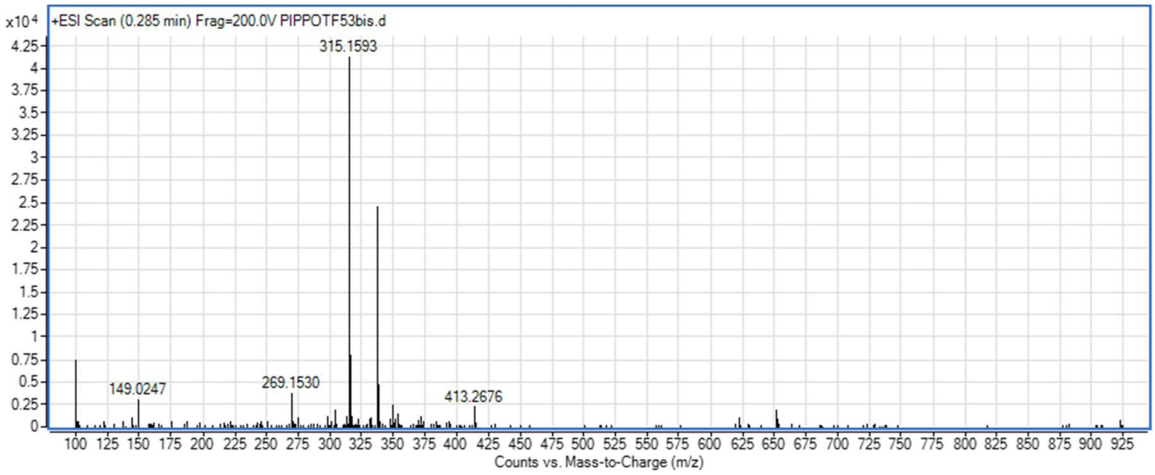

Figure S16: HR ESIMS spectrum (positive mode) of flavuglaucin C (**8**)

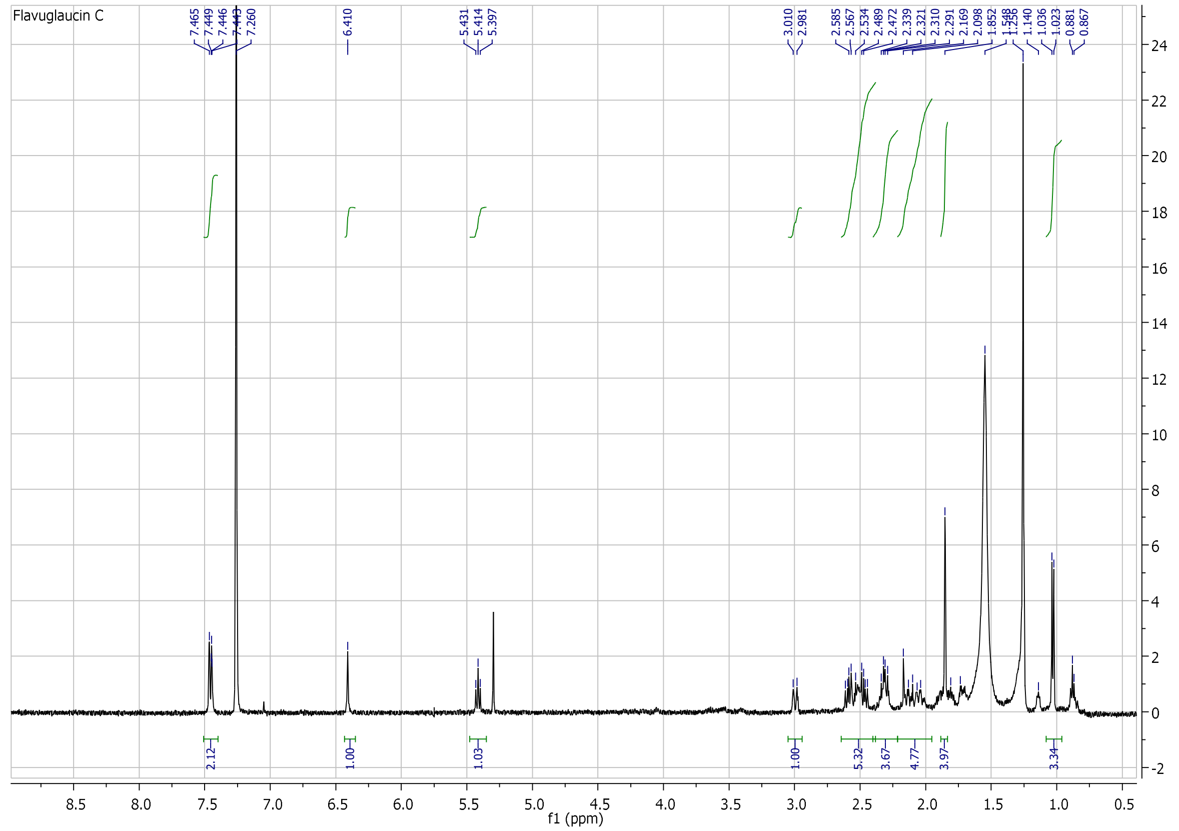

Figure S17: ^{1}H NMR spectrum (500 MHz, $CDCl_3$) of flavuglaucin C (**8**)

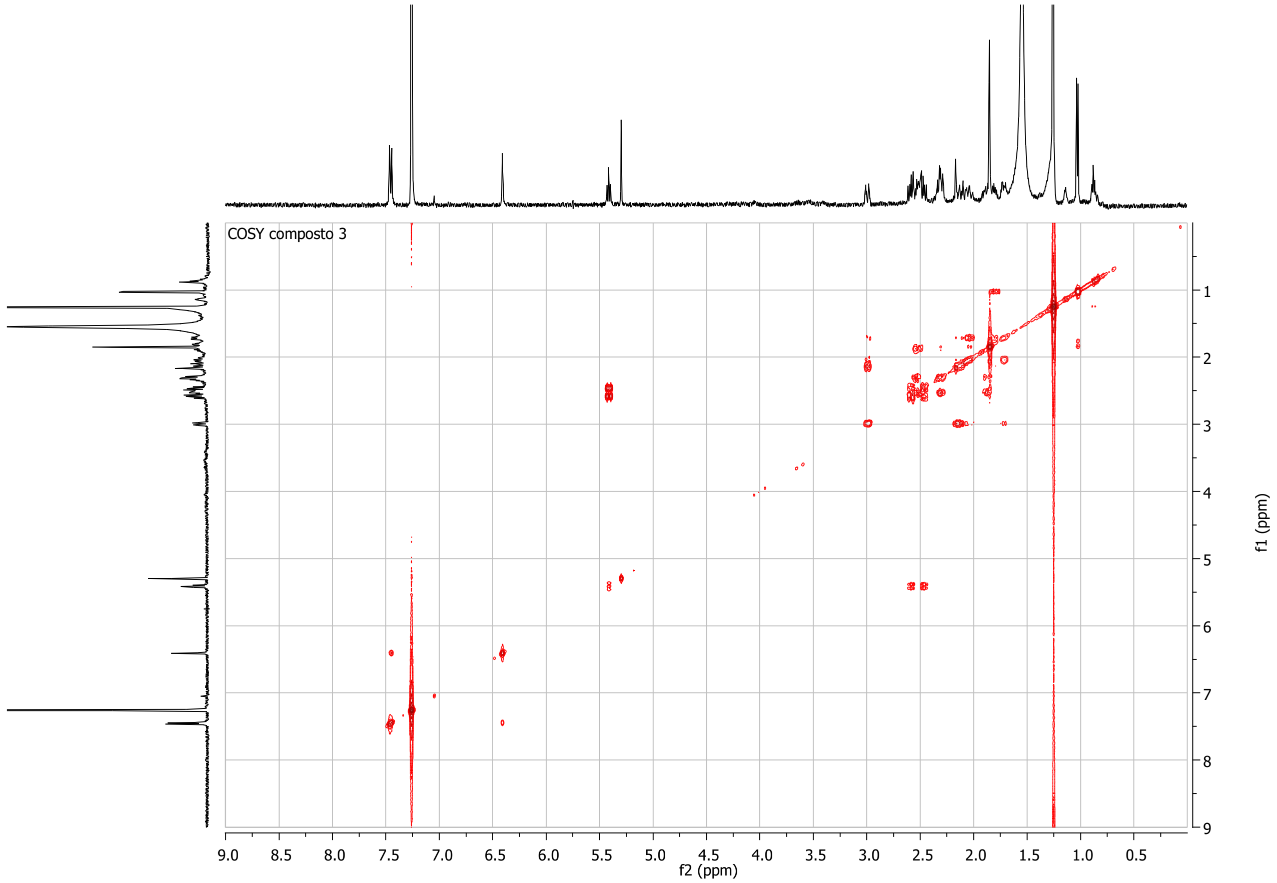

Figure S18: COSY spectrum (500 MHz $CDCl_3$) of flavuglaucin C (**8**)

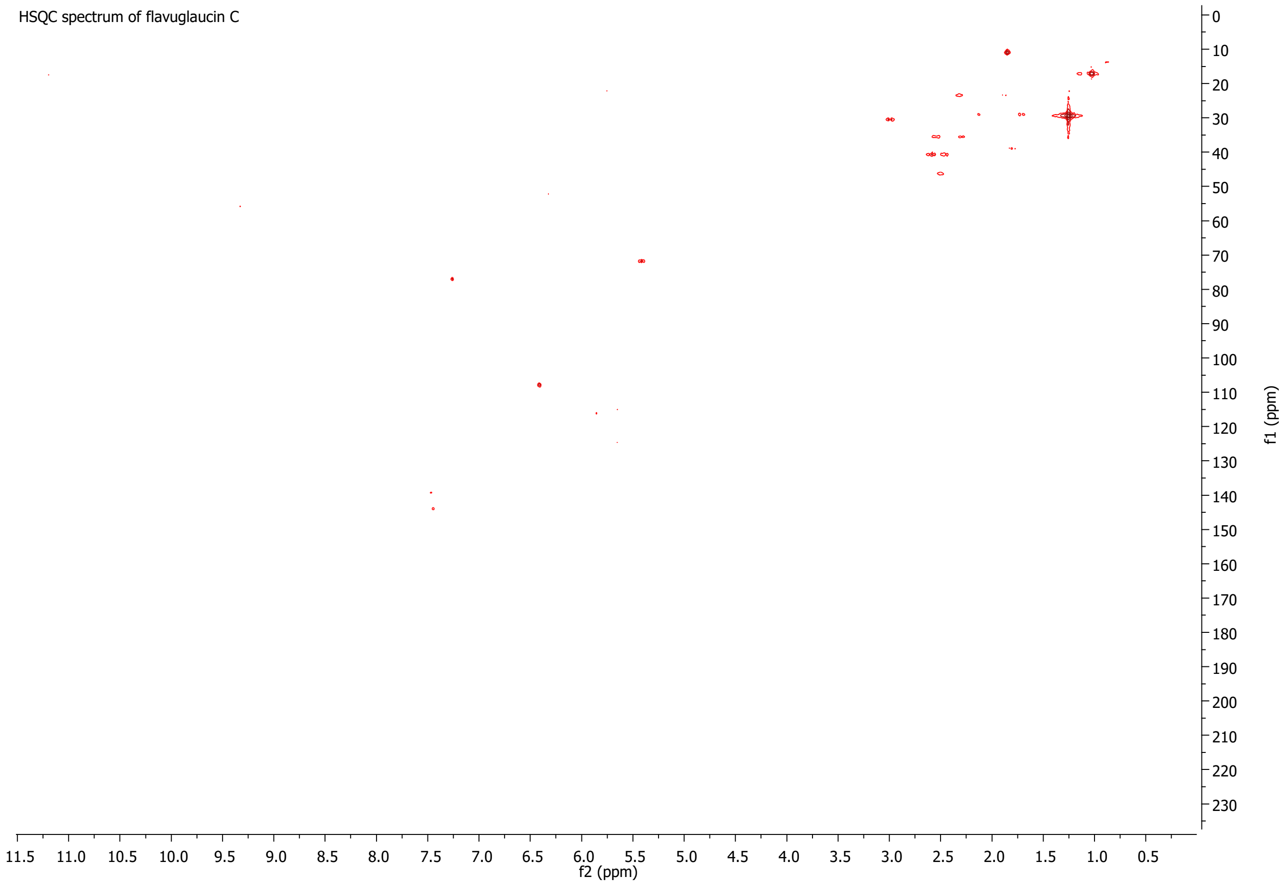

Figure S19: HSQC spectrum (500 MHz $CDCl_3$) of flavuglaucin C (**8**)

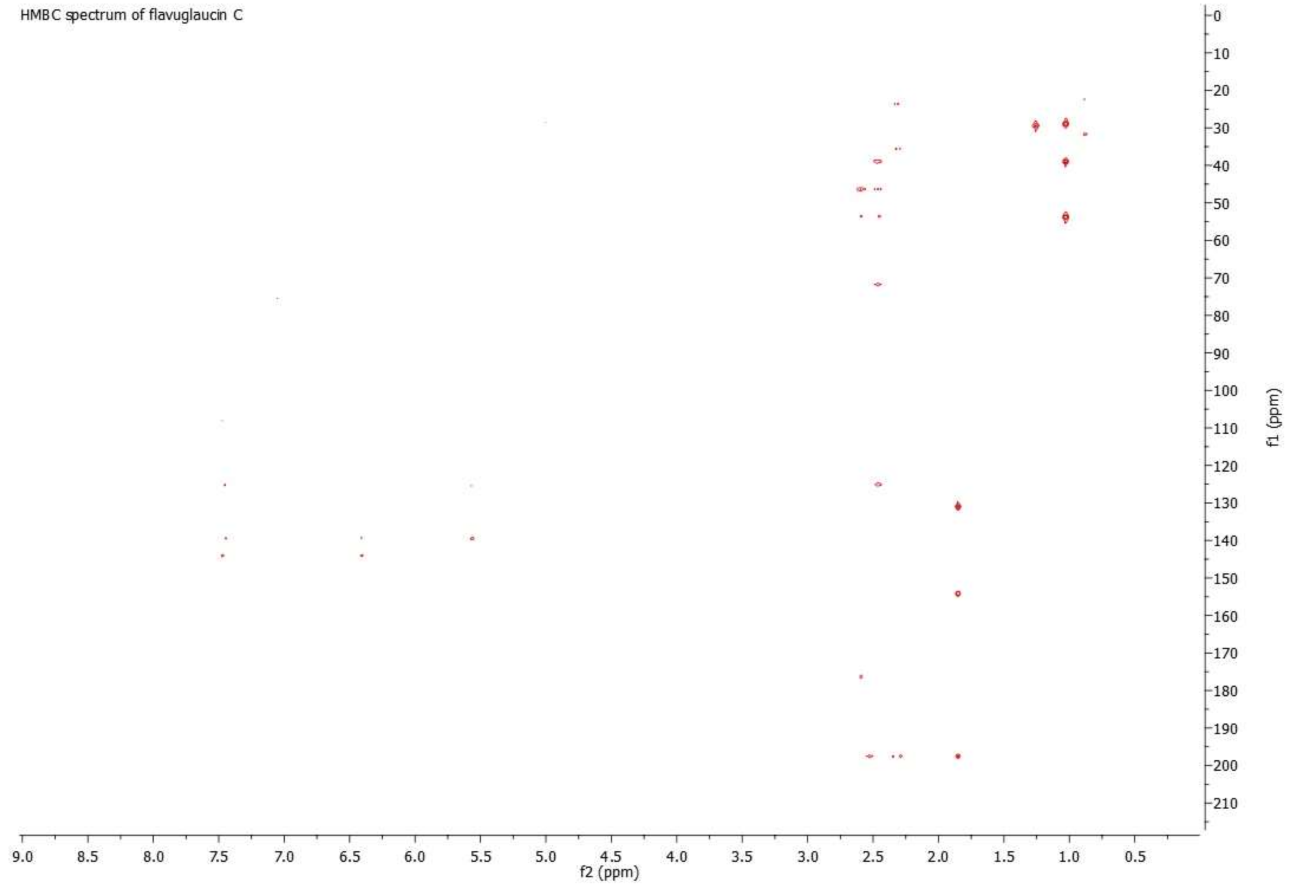

Figure S20: HMBC spectrum (500 MHz $CDCl_3$) of flavuglaucin C (**8**)

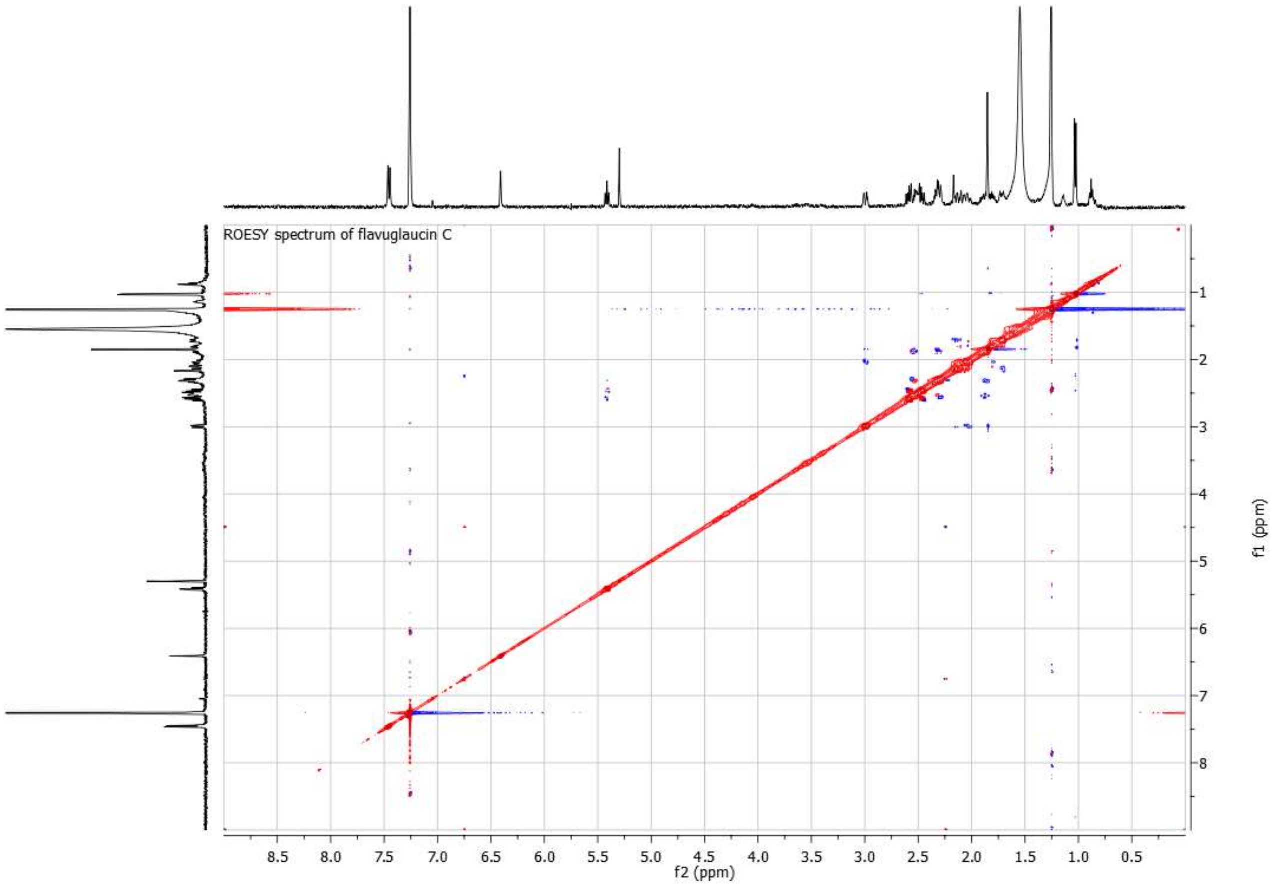

Figure S21: ROESY spectrum (500 MHz $CDCl_3$) of flavuglaucin C (**8**)
